# *Campylobacter jejuni* Colonization Is Associated with a Dysbiosis in the Cecal Microbiota of Mice in the Absence of Prominent Inflammation

**DOI:** 10.1371/journal.pone.0075325

**Published:** 2013-09-16

**Authors:** Abdul G. Lone, L. Brent Selinger, Richard R. E. Uwiera, Yong Xu, G. Douglas Inglis

**Affiliations:** 1 Agriculture and Agri-Food Canada Research Centre, Lethbridge, Alberta, Canada; 2 Department of Biological Sciences, University of Lethbridge, Lethbridge, Alberta, Canada; 3 Department of Agricultural, Food and Nutritional Science, University of Alberta, Edmonton, Alberta, Canada; University of California, Riverside, United States of America

## Abstract

**Background:**

*Campylobacter jejuni* causes enterocolitis in humans, but does not incite disease in asymptomatic carrier animals. To survive in the intestine, *C. jejuni* must successfully compete with the microbiota and overcome the host immune defense. *Campylobacter jejuni* colonization success varies considerably amongst individual mice, and we examined the degree to which the intestinal microbiota was affected in mice (i.e. a model carrier animal) colonized by *C. jejuni* at high relative to low densities.

**Methods:**

Mice were inoculated with *C. jejuni* or buffer, and pathogen shedding and intestinal colonization were measured. Histopathologic scoring and quantification of mRNA expression for α-defensins, toll-like receptors, and cytokine genes were conducted. Mucosa-associated bacterial communities were characterized by two approaches: multiplexed barcoded pyrosequencing and terminal restriction fragment length polymorphism analysis.

**Results:**

Two *C. jejuni* treatments were established based on the degree of cecal and colonic colonization; *C. jejuni* Group A animals were colonized at high cell densities, and *C. jejuni* Group B animals were colonized at lower cell densities. Histological examination of cecal and colonic tissues indicated that *C. jejuni* did not incite visible pathologic changes. Although there was no significant difference among treatments in expression of mRNA for α-defensins, toll-like receptors, or cytokine genes, a trend for increased expression of toll-like receptors and cytokine genes was observed for *C. jejuni* Group A. The results of the two methods to characterize bacterial communities indicated that the composition of the cecal microbiota of *C. jejuni* Group A mice differed significantly from *C. jejuni* Group B and Control mice. This difference was due to a reduction in load, diversity and richness of bacteria associated with the cecal mucosa of *C. jejuni* Group A mice.

**Conclusions:**

High density colonization by *C. jejuni* is associated with a dysbiosis in the cecal microbiota independent of prominent inflammation.

## Introduction

*Campylobacter jejuni* is a curved gram-negative motile bacterium, which is a common cause of foodborne enteritis in humans in the developed world [1,2,3]. Campylobacteriosis is characterized by fever, abdominal pain, watery to bloody diarrhea. In some instances, infected individuals may subsequently develop reactive arthritis, neurological disorders, or inflammatory bowel disease [4,5,6]. The bacterium readily colonizes a wide variety of animals asymptomatically (e.g. wildlife and livestock), and these animals may serve as a reservoir of infectious cells to humans [3,7,8]. Although *C. jejuni* is not considered to be a normal constituent of the intestinal microbiota of humans, a large number of asymptomatic humans were positive for the bacterium in developing countries [9]. Furthermore, a high number of individuals may be colonized by *C. jejuni* without exhibiting any clinical symptoms during outbreaks of the disease [1,10,11].

The mammalian intestinal tract harbours large numbers of bacterial cells (^≈^100 trillion) and hundreds of different species which are thought to prevent colonization and growth of many intestinal pathogens including *C. jejuni*, although the mechanisms are poorly understood at present [6,12]. This phenomenon of microbiota-imparted resistance to colonization against pathogens is commonly known as “colonization resistance”. In addition to colonization resistance, re-establishing eubiosis following host damage is also essential for pathogen clearance [13]. To successfully colonize a host, some intestinal pathogens alter the composition of host microbiota and this change in the microbiota is believed to be primarily due to the host inflammatory response [6,14,15,16,17]. However, an alteration in microbiota composition by means other than host inflammation (e.g. due to antibiotic administration or physiological stress) also facilitates colonization by intestinal pathogens [18,19,20,21]. The mechanisms by which the microbiota prevents colonization by bacterial pathogens is believed to be due the production of inhibitory substances (e.g. bacterial metabolites, bacteriocins), depletion of nutrients (which will be efficient in highly diverse and rich bacterial communities), and/or stimulation of the host immune system [22]. Conversely, it is also possible that a bacterial pathogen like *C. jejuni*, which readily colonizes the intestines of a diverse number of non-human mammals and avian species at high cell densities without inciting prominent inflammation [7,23,24], is able to affect the microbiota composition to allow it to persist in these hosts.

It is not currently known whether *C. jejuni* influences the composition of microbiota to facilitate colonization in asymptomatic animals or whether the microbiota from particular animal species is naturally amenable to high density colonization by *C. jejuni*. To examine the association between *C. jejuni* colonization in relation to the intestinal microbiota in an asymptomatic host, we chose mice as a mammalian model. *Campylobacter jejuni* typically colonizes mice without causing any illness [14,25,26,27,28,29,30], and like humans, mice are not consistently colonized by *C. jejuni*. However, once mice become colonized by *C. jejuni*, the bacterium can remain in high numbers within the intestine for prolonged periods similarly to other mammals and birds [14,29]. Furthermore, the intestinal microbiota of mice is often used as a model for the human enteric microbiota [22]. Ascertaining the degree to which the intestinal microbiota is altered subsequent to colonization by *C. jejuni* in an asymptomatic host is an important step toward elucidating the mechanisms by which this important enteric pathogen colonizes the intestines of mammals. We hypothesized that the enteric microbiota will differ in mice colonized by *C. jejuni* at high densities in the absence of inflammation.

## Materials and Methods

### Ethics statement

The study was carried out in strict accordance with the recommendations specified in the Canadian Council on Animal Care Guidelines. The project was reviewed and approved by the Lethbridge Research Centre (LRC) Animal Care Committee (Animal Use Protocol Review 0703) and the LRC Biosafety and Biosecurity Committee before commencement of the research. The stool sample of the human infected by *C. jejuni* NCTC 11168 was donated by the afflicted individual, and written informed consent was provided by the infected individual to isolate *C. jejuni* from their stool sample, and to genotype and utilize the recovered *C. jejuni* isolates in subsequent research.

### Animals

Parent mice (C57/6J) were obtained from Jackson Laboratories (Bar Harbor, ME) and the mice were bred and reared using standard protocols. Twenty-two F_1_ offspring at *ca.* 5 weeks of age were used in the experiment, and individual mice were randomly assigned to treatments. Mice were individually maintained in ventilated cages (Techniplast, Exton, PA), and provided with autoclaved feed (Prolab RM 3500, LabDiet, ON, Canada); animals were permitted to feed and drink *ad libitum*.

### Inoculum

*Campylobacter jejuni* NCTC 11168 passed through a human was used. A human who had been working with NCTC 11168 developed severe enteritis, *C. jejuni* was isolated from a stool sample obtained from the afflicted individual, and it was genotyped using a 40 locus comparative genomic fingerprint method [31] which showed that it possessed an identical fingerprint pattern to NCTC 11168 (data not presented). Further, the strain of *C. jejuni* recovered from the stool sample was whole genome sequenced and compared to the whole genome sequence of the NCTC 11168 (i.e. the strain with which the human had been working), and was confirmed to be the same strain (data not presented). To produce inoculum, *C. jejuni* was grown in a microaerobic environment (10% CO_2_, 3% H_2_, 5% O_2_, 82% N_2_) at 37°C on Columbia agar (Oxoid, Nepean, ON) supplemented with 5% sheep blood for 16 hr. Cells were harvested in phosphate buffered saline (pH 7.2; PBS), and cell densities were adjusted to a final optical density (OD_600_) of 0.5. This OD corresponded to a cell density of between 10^8^ to 10^9^ colony forming units (CFU) per ml. Cells were maintained on ice until used.

### Inoculation of mice

To confirm that mice were free of *C. jejuni*, freshly voided feces was collected, genomic DNA was extracted, and *C. jejuni*-specific PCR targeting the *map*A gene was conducted [32]. Mice were arbitrarily divided into two groups. The first group consisted of 14 animals and they were inoculated with *C. jejuni* (*C. jejuni* group). The second group consisted of six animals that were inoculated with buffer alone (i.e. Control). The *C. jejuni*-inoculated group contained more animals to ensure that a sufficient number of animals were colonized by the bacterium. Mice were gavage inoculated with 100 µl of the suspension of *C. jejuni* or PBS alone. Mice were inoculated within 30 min of the collection of *C. jejuni* cells. To confirm densities of viable *C. jejuni* cells, inoculum was diluted in a 10-fold dilution series, 100 µl of each dilution was spread in duplicate onto Karmali agar (Oxoid), cultures were incubated at 37°C in a microaerobic atmosphere, and the number of *C. jejuni* colonies were counted at the dilution yielding 30 to 300 CFU after 48 and 72 hr of incubation. Aliquots of the inoculum were also examined microscopically for the presence of highly motile *C. jejuni* cells. After inoculation, mice were observed at least once per day for behavioural signs of disease, weight loss, dehydration, fecal consistency, or any other clinical signs of disease.

### Collection of feces and weighing of animals

Freshly voided fecal pellets were collected from each mouse 0.5, 7, 14, and 21 days post-inoculation (p.i.). Each sample was weighed, homogenized in 1 ml of PBS, the homogenate diluted in a 10-fold dilution series, and 100 µl of each dilution was spread in duplicate onto Karmali agar containing selective supplement SR167 (Oxoid). Cultures were maintained at 37°C in a microaerobic atmosphere, and the number of CFU determined as described above. Animals were also weighed each time feces were collected. Data were analyzed using the MIXED procedure of SAS (SAS Institute Inc., Cary, NC), and collection time was treated as a repeated measure. The appropriate covariance structure was utilized according to the lowest Akaike’s Information Criterion.

### Tissue collection and gross pathology

Mice were humanely euthanized 21 days p.i. Mice were first anesthetized with isofluorane gas (Halocarbon Products Corporation, River Edge, NJ) and then euthanized with an overdose of CO_2_. Immediately after death, a midline incision was made, and the gastrointestinal tract (GIT) and associated tissues were exteriorized and observed for gross abnormalities (e.g. increased intestinal wall thickness, enlarged mesenteric lymph node). The entire stomach, the distal jejunum, and the entire ileum, cecum, and colon were aseptically removed from each mouse. The distal portion of each tissue segment (^≈^0.5 cm) was removed and processed for RNA extraction, DNA extraction, and histopathology. Residual ingesta was removed by gently submerging the tissues in sterile phosphate buffered saline, and bacteria retained after the gentle rinse were considered “mucosa-associated”. Each tissue type (^≈^10 mg) was aseptically removed for DNA extraction, placed in 2.0 ml tubes on ice, and samples stored at -20°C (within 30 min of animal euthanization) until processed. For RNA extraction, cecal tissues were immediately immersed in RNAlater^TM^ (Life Technologies, Burlington, ON) in 2.0 ml tubes (within 10 min of animal euthanization), and stored at -80°C until processed. For histopathologic examination, tissue segments were placed in 10% phosphate buffered formalin (Surgipath Canada Inc., Winnipeg, MB), gently agitated to remove ingesta, transferred to histological cassettes, and submerged in a fresh solution of phosphate buffered formalin. The proximal segment of each tissue segment was opened longitudinally, and examined closely for gross pathologic changes (e.g. congestion, presence of blood or abnormal quantities of mucus) after tissues had been collected for DNA and RNA extraction, and histopathologic examination.

### Histopathology

Cecal and colonic samples were maintained in 10% buffered formalin for a maximum of 2 weeks. Tissues were dehydrated with ethanol and Histoclear (Fisher Scientific Inc, Toronto, ON, Canada), and paraffinized with Paraplast Plus (Fisher Scientific Inc.) for 2 hr at 60°C in a vacuum oven. Tissues were embedded using a Shandon Histocentre III (Fisher Scientific Inc.), sectioned (^≈^4 µm) using a Finesse 325 microtome (Fisher Scientific Inc.), and sections were placed on Superfrost Plus Gold slides (Fisher Scientific Inc.). Sections were de-paraffinized with xylene, stained with hematoxylin and eosin following a standard protocol, and examined for congestion, mucosal necrosis, neutrophils, macrophages and lymphocyte infiltration, goblet cell size and number, tissue congestion, lympholysis, and fibrosis. Histological inflammation scoring was performed in a “blinded” fashion (i.e. as to treatment) by a veterinary pathologist (RREU), with scoring criteria adapted from previously described methods [33,34]. With the exception of goblet cell size and number (scored 0 to 3), cecal and colonic tissues were graded from 0 (normal) to 4 (marked changes). In addition, a total score was calculated by summing scores of mucosal necrosis, neutrophil, macrophage and lymphocyte infiltration, goblet cell size and number, and fibrosis. As the data was categorical, treatments were compared non-parametrically using the NPAR1WAY procedure of SAS (SAS Institute Inc.) with the Wilcoxon rank-sum test. Total histopathologic scores were categorized as no effect to negligible changes (score of 0 to 4), mild to moderate changes (score of 5 to 9), and marked changes (score of 10 to 19).

### RNA extraction and quantification of α-defensin, toll-like receptor and cytokine mRNA expression

Total RNA was extracted from cecal tissue using the RNeasy mini protocol for isolation of total RNA from animal tissues and the RNeasy Mini Kit (Qiagen Inc.) according to the manufacturer’s recommendations. RNA was checked for quality and quantity by electrophoresis. Any contaminating DNA was removed by DNase digestion (Qiagen Inc.). RNA was reverse transcribed into cDNA using the QuantiTect Reverse Transcription Kit (Qiagen Inc.). An in-house qPCR array (384 well format) was used to quantify the mRNA expression of the following genes: cryptdin peptide (cryptdin) 4; cryptdin 5; cryptdin 20; toll-like receptor (TLR) 2; TLR4; TLR5; TLR9; interferon (INF)-γ; interleukin (IL)-1β; IL-4; IL-5; IL-6; IL-10; IL-17A; IL-22; tumor necrosis factor (TNF)-α; and TNF-β. The housekeeping genes Actb (β-actin), B2m (β-2-microglobulin), GusB (β-glucuronidase), Ldha16a (lactate dehydrogenase A), and Ppia (peptidylprolyl isomerase A) were evaluated. Of these genes, three stably expressed housekeeping genes (Actb, B2m, and Ppia) were selected via geometric averaging [35]. Published primers for IL-10 were used [36]. All other primers were designed using Primer 3 and reference gene sequences within the National Center for Biotechnology Information (NCBI) website. Primers were designed to produce a single amplicon between 140-160 base pairs (bp) in size, and to have a Tm of 60°C. Quantitech SYBRgreen (Qiagen Inc.) real-time PCR was completed using an ABI 7900HT thermocycler (Applied Biosystems, Burlington, ON). PCR conditions were 95°C for 15 min, followed by 40 cycles of 94°C for 15 sec, 58°C for 30 sec, and 72°C for 30 sec. Reverse transcription and genomic DNA controls were included. A four point five-fold standard curve for each gene was included for the calculation of amplification efficiencies. Following amplification, melt curve analysis was conducted to confirm amplification specificity. All reactions were run in triplicate, and the mean value of the observations was used for analysis. Normalized gene expression was calculated using qbasePLUS (Biogazelle, Zwijnaarde, Belgium) based on geNorm and qBase quantification models [35,37], and log_10_-transformed data were analyzed using the one-way analysis of variance feature within the program.

### DNA extraction

DNA was extracted from cecal tissue using a RTP Bacteria DNA Mini Kit (Invitek, Berlin, Germany), according to the manufacturer’s instructions. Concentrations of DNA were quantified spectrophotometrically. DNA was stored at -80°C until utilized.

### Intestinal colonization by *Campylobcter* *jejuni*

Densities of *C. jejuni* and total bacteria associated with mucosa of the stomach, jejunum, ileum, distal colon were determined by quantitative PCR targeting the *map*A and 16S rRNA gene, respectively; PCR efficiency, optimum primer concentration, and dynamic range were determined in advance. DNA was diluted to reduce the concentration of any PCR inhibitors present. The SYBR Green-based standard curve method for quantification of DNA was carried out using Power SYBR® Green PCR (Life Technologies). Each 20 µl PCR reaction contained 2 µl of DNA (20-50 ng), 10 µl of the 2X Power SYBR® Green PCR Master Mix, and 200 nmol of each of the forward and reverse primers. For the quantification of *C. jejuni*, the QCjmapANF and QCjmapANR primers were used [38]. For quantification of total bacteria, the HDA1 and HDA2 primers were used [39]. Standard curves were established using genomic DNA from *C. jejuni* or *Escherichia coli* (ATCC 25922). DNA copy number varied from 10^1^ to 10^7^; as there are seven copies of the 16S rRNA gene in *E. coli* ATCC 259229, the number of 16S rRNA gene copies in the standard curve were adjusted accordingly. Samples were amplified as follows: one cycle at 95°C for 10 min; and 40 cycles at 95°C for 15 sec, and at 60°C for 60 sec. A Stratagene Mx 3005 (Stratagene Products, La Jolla, CA) was used. All reactions were run in triplicate, and the mean value of the observations was used for analysis. The number of bacteria was expressed as copy number per gram of tissue. For all reactions, melt curve analysis was conducted to confirm amplification specificity. Data were analyzed using a one-way analysis of variance using the MIXED procedure of SAS (SAS Institute Inc.). In conjunction with a significant F-test, the lsmeans function of SAS was used to compare treatments.

### Sequence-Based Bacterial Community Analysis

The basic bacterial tag-encoded FLX 454-pyroseqencing (bTEFAP) procedure was performed as described previously [20,40,41]. Briefly, DNA from each cecal sample was diluted to a final concentration 2 ng µl^-1^. An initial 30-cycle PCR was performed to amplify a 512 bp region of the 16S rRNA gene spanning variable regions V1 to V3 using Gray28F (5′-GAGTTTGATCNTGGCTCAG -3’) and Gray519r (5′-GTNTTACNGCGGCKGCTG-3’) [41] with HotStar high fidelity Taq polymerase (Qiagen, Valencia, CA). The resulting PCR product was used as template in a second PCR reaction with fusion primers [42]. PCR products from different samples were barcoded and bTEFAP was completed using a Roche 454 FLX instrument (Roche, Nutley, NJ) with Titanium reagents at the Research and Testing Laboratory (Lubbock, TX). Raw data was processed to remove sequences less than 200 bp, and sequences containing homopolymers greater than 8 bp, mismatches in the barcode or primer, one or more ambiguous bases, or an average quality score below 30 over a moving window of 50 bp. The remaining sequences were aligned to the SILVA-based bacterial reference alignment [43] using of Needleman-Wunsch algorithm [44]. Potential chimeric sequences were removed using UCHIME [45], sequencing noise was reduced by applying a preclustering step, and sequences assigned to the Cyanobacteria lineage were removed. The cleaned pyrotag data was processed using the Quantitative Insights Into Microbial Ecology (QIIME) pipeline [46]. The ‘uclust’ method within QIIME was used to cluster Operational Taxonomic Units (OTUs) at a 97% similarity level, and the RDP classifier was applied to classify OTU’s at an 80% confidence level. Richness, Chao1 estimates, Shannon’s index, and phylogenetic diversity were calculated by sample and treatment. For calculation of alpha diversity metrics, the lowest number of sequences per sample within individual treatment groups was used; 2892, 1916, and 2057 for *C. jejuni* Group A, *C. jejuni* Group B, and Control samples, respectively. For pairwise t-tests, data was normalized to 1916 for all samples. The heat map was generated for each sample by treatment; only OTUs for which ten or more sequences were observed were included in the heat map. Principal coordinate cluster analysis (PCoA) was conducted using the Unifrac distance metric on weighted (normalized abundance values) and unweighted datasets that were subsampled to an even depth. The R package (Available: http://www.r-project.org/. Accessed 2013 Jul 09) was used for data visualization. To statistically compare community compositions between treatments, pairwise analysis of similarity (ANOSIM) was performed (i.e. weighted and unweighted) using Vegan (999 permutations) [47]. Sequences were accessioned in GenBank (NCBI) under: SRR933603 (A1); SRR933605 (A2); SRR933606 (A3); SRR933608 (A4); SRR933609 (A5); SRR934203 (A6); SRR934204 (B1); SRR934205 (B2); SRR934206 (B3); SRR934207 (B4); SRR934208 (B5); SRR934209 (B6); SRR934210 (C1); SRR934211 (C2); SRR934212 (C3); SRR934213 (C4); SRR934214 (C5); and SRR934215 (C6).

### Fingerprint-based bacterial community analysis

The basic terminal restriction fragment length polymorphism (T-RFLP) and analysis protocol described by Costa et al. [48] was used. The primers 27f and 1492r [49] were used to amplify the 16S rRNA gene in 10 ng of cecal DNA. The forward primer was labeled with FAM (FAM27f). Each reaction consisted of 2 µl of genomic DNA (^≈^10 ng), 2.0 µl of 1X PCR buffer, 0.1 µl of each deoxynucleoside triphosphate (0.2 mM), 2.0 µl of acetylated bovine serum albumin (BSA; Promega, Madison, WI; 0.1 µg µl^-1^), 0.1 µl of Taq DNA polymerase (Qiagen, Inc.; 5 units µl^-1^), 1.0 µl each of the bacterial primers (0.5 µM), and 11.5 µl Optima water (Fisher Scientific, Ottawa, ON). PCR conditions were 95°C for 15 min, 25 cycles consisting of 94°C for 30 sec, 53°C for 60 sec, and 72°C for 60 sec, and a final extension period at 72°C for 10 min. All PCR reactions were performed in triplicate, and pooled before restriction digestion. All amplicons were electrophoresed in a 1% TAE agarose gel relative to a 100 bp DNA ladder (Promega). The target amplicon of ^≈^1500 bp was purified using a QIAquick PCR purification Kit (Qiagen, Inc.), and DNA concentrations were quantified using a TD 360 Mini Fluorometer (Turner Designs, Sunnyvale, CA) and TNE / Hoescht dye buffer. If required, DNA concentrations were also quantified by agarose gel electrophoresis. Concentrations of DNA in all samples were standardized to 25 ng µl^-1^ using Optima water. Restriction digestions were carried out in duplicate in a mixture containing 75 ng of the purified PCR product, 3 units of HaeIII (Life Technologies), 2.5 µl of enzyme buffer, and Optima water to a final volume of 25 µl. Samples were incubated at 37°C for 2 hr in the dark, and ethanol precipitation was performed to stop the reaction by adding 50 µl of 95% ethanol and 2 µl of sodium acetate (pH 5.2) to each sample. Samples were incubated for 20 min at 20°C, and centrifuged for 20 min (13,200 x g) to pellet DNA. Nucleic acids were washed by adding 500 µl of 70% ethanol, followed by centrifugation at 13,200 x g for 5 min. After ethanol precipitation, samples were air dried overnight in the dark, re-suspended in 9.25 µl of Hi Di formamide (Applied Biosystems Canada, Streetsville, ON) and 0.25 µl of LIZ600 size standard marker (Applied Biosystems Canada), denatured at 95°C for 3 min, and immediately placed on ice. Fluorescent labeled terminal restriction fragments (T-RFs) were separated in POP7 polymer using a 3130 Genetic Analyzer (Applied Biosystems Canada), and analyses were performed on T-RFs ranging in size from 50 to 580 bp covering V1 to V3 of the 16S rRNA gene. Electropherograms were analyzed using GeneMapper software version 4.0 with the Local Southern size calling method (Applied Biosystems Canada) as described previously [48]. Euclidean distance, and Pearson and Dice coefficients were calculated to cluster animals into groups, and the clusters were linked together by unweighted pair-group using the centroid average (UPGMA) and Ward’s method within the Bionumerics software (Applied Maths, Austin, TX). The statistical significance of each group was tested by comparing between group similarities with randomization tests using 1000 iterations (Applied Maths Inc.) [48]. To further explore the composition of bacterial communities, non-metric multi-dimensional scaling (NMS) was applied using SAS (SAS Institute Inc., Cary, NC), and three dimensional NMS plots were graphed using SigmaPlot (Systat Software Inc., Chicago, IL).

## Results

### Two groups of mice were observed based on Intestinal colonization by *Campylobacter jejuni*

The group of mice gavaged with *C. jejuni* consisted of more animals than the *C. jejuni*-free Control treatment to account for inconsistent intestinal colonization by *C. jejuni* amongst individual mice [30,50]. Four animals in the *C. jejuni*-inoculated group were either not colonized or were colonized at very low densities (<100 CFU g^-1^ of feces), and *C. jejuni* was successfully cleared by these animals during the experimental period. Since we were interested in ascertaining the effects of *C. jejuni* colonization on the host microbiota over longer periods (i.e. 21 days), we excluded these four animals from subsequent analysis. In the remaining inoculated mice, *C. jejuni* was detected in the feces of all individuals over the 21-day experimental period (Table 1). The *C. jejuni* inoculated group was divided into two distinct groups based on colonization density (i.e. *C. jejuni* Group A, and *C. jejuni* Group B). Campylobacter jejuni Group A mice shed significantly larger numbers of *C. jejuni* cells (^≈^4 orders of magnitude) in feces than did *C. jejuni* Group B mice throughout the experimental period (Table 1). The Group A mice also had higher numbers (^≈^2 orders of magnitude) of *C. jejuni* cells associated with mucosa within their ceca than *C. jejuni* Group B mice (Table 2). The same pattern of *C. jejuni* colonization was observed in colon (Table 2). Based on colonization patterns, the following three treatment groups were established: (1) *C. jejuni* Group A consisted of six mice colonized by high densities of *C. jejuni*; (2) *C. jejuni* Group B consisted of six mice colonized by lower densities of the bacterium; and (3) the Control consisted of six animals devoid of *C. jejuni* (18 mice total).

**Table 1:** Log_10_ *C. jejuni* CFU g^-1^ of mice feces (mean ± standard error of the means).

**Group**	**7 days p.i.**	**14 days p.i.**	**21 days p.i.**
*C. jejuni* Group A^a^	8.91 ± 0.09 a^b^	8.28 ± 0.36 a	7.88 ± 0.43 a
*C. jejuni* Group B	4.72 ± 0.26 b	4.41 ± 0.17 b	3.16 ± 0.28 b
Control	0.0	0.0	0.0

a Group A mice and Group B mice were inoculated with *C. jejuni*, whereas Control mice were gavaged with buffer alone.

b Means not followed by the same letter within columns differ (P<0.05).

**Table 2:** Copy number (Log_10_ g^-1^) of mucosa-associated *C. jejuni* in ceca and colons, and total bacteria in ceca of mice (mean ± standard error of the means).

**Group**	**Cecum – *C. jejuni***	**Colon – *C. jejuni***	**Cecum - Total**
*C. jejuni* Group A^a^	8.80 ± 0.07 a^b^	7.07 ± 0.11 a	10.43 ± 0.11 a
*C. jejuni* Group B	6.42 ± 0.19 b	5.41 ± 0.15 b	10.78 ± 0.12 b
Control	0.0	0.0	10.79 ± 0.06 b

a Group A mice and Group B mice were inoculated with *C. jejuni*, whereas Control mice were gavaged with buffer alone.

b Means not followed by the same letter within columns differ (P≤0.05).

### Absence of inflammation and no impact on growth of mice colonized by *Campylobacter jejuni*

**Figure 1:**
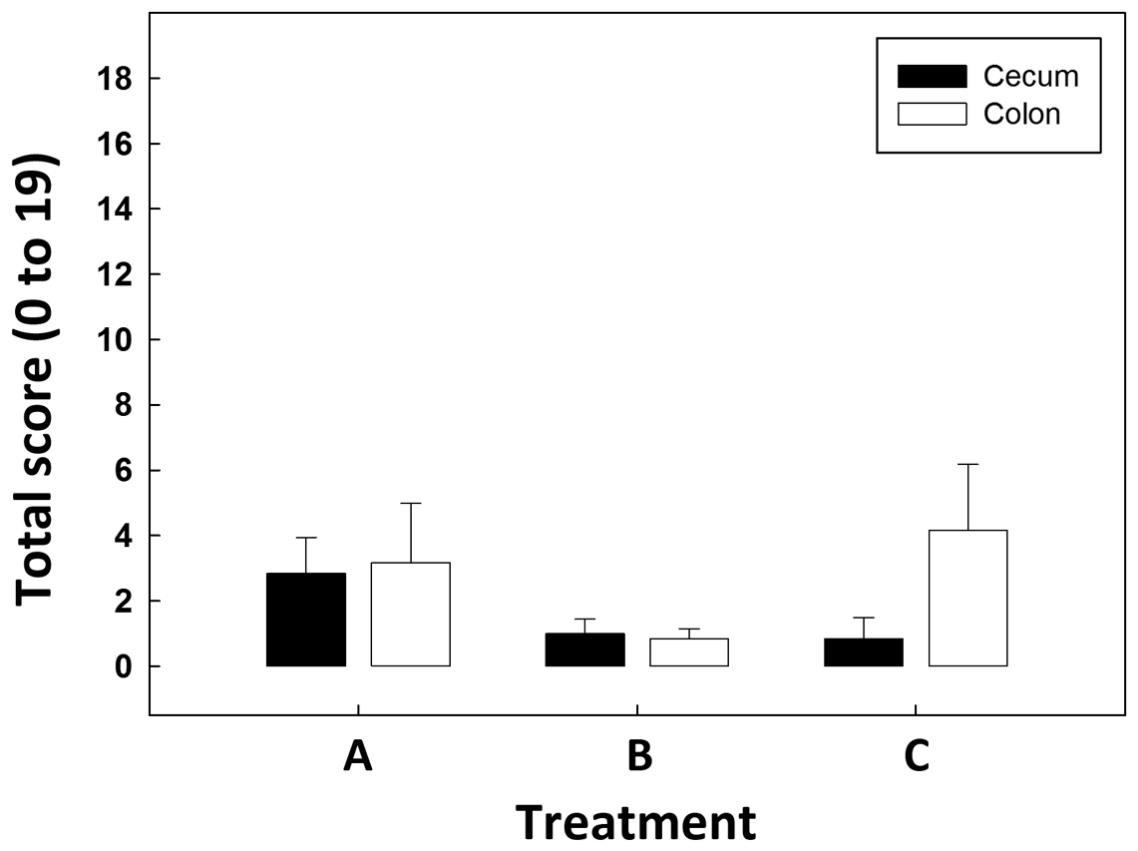
Histopathologic changes. Mean histological scores of cecal and colonic tissues for combined necrosis, neutrophils, macrophages and lymphocytes, and fibrosis, and goblet cell size and number. Treatments are: (A) *C*. *jejuni* Group A (8.8 log_10_ copy number of *C*. *jejuni* g^-1^ of cecal tissue); (B) *C*. *jejuni* Group B (6.4 log_10_ copy number of *C*. *jejuni* g^-1^ of cecal tissue); and (C) control (not inoculated with *C*. *jejuni*). Vertical lines associated with histogram bars are standard error of the means (n=6). There were no significant differences for cecal (P≥0.20) or colonic (P≥0.34) tissues among treatments. Scores were categorized as no effect to negligible changes (score of 0 to 4), mild to moderate changes (score of 5 to 9), and marked changes (score of 10 to 19).

No clinical signs of illness (e.g. diarrhea, malaise), increased mucus production, intestinal distension, or gross evidence of inflammation or lesions were observed in any of the mice regardless of whether they were inoculated with *C. jejuni*. Microscopically, very low scores (≤1) were observed for congestion, changes in goblet cell size and number, tissue congestion, and lympholysis (data not shown). There were no significant differences (P≥0.12) in mucosal necrosis, neutrophil infiltration, macrophage and lymphocyte infiltration, or fibrosis in the cecum or colon among the three treatments (data not shown). Furthermore, there was no difference (P≥0.20) in total histopathology scores among treatments for either location (Figure 1). Mean total histopathology scores were ≤2.8 ± 1.1 and ≤4.2 ± 2.0 for the cecum and distal colon, respectively; a score of 4.0 or less indicates negligible changes. In addition, no significant difference (P>0.05) was observed in growth, measured as weekly increase in body weight among treatments (Table 3).

**Table 3:** Weekly increase in body weight (g) of mice (mean ± standard error of the means).

**Group^a^**	**7 days p.i.**	**14 days p.i.**	**21 days p.i.**
Group A	2.39 ± 0.41 a^b^	1.56 ± 0.63 b	0.82 ± 0.23 c
Group B	2.60 ± 0.34 a	1.18 ± 0.33 b	0.71 ± 0.19 c
Control	2.74 ± 0.41 a	0.92 ± 0.29 b	0.64 ± 0.18 c

a Group A and Group B mice were inoculated with *C. jejuni*, whereas Control mice were gavaged with buffer alone.

b Means not followed by the same letter within columns differ (P<0.05).

### Cecal colonization by *C. jejuni* did not significantly affect α-defensin, toll-like receptor, or cytokine mRNA expression

**Figure 2:**
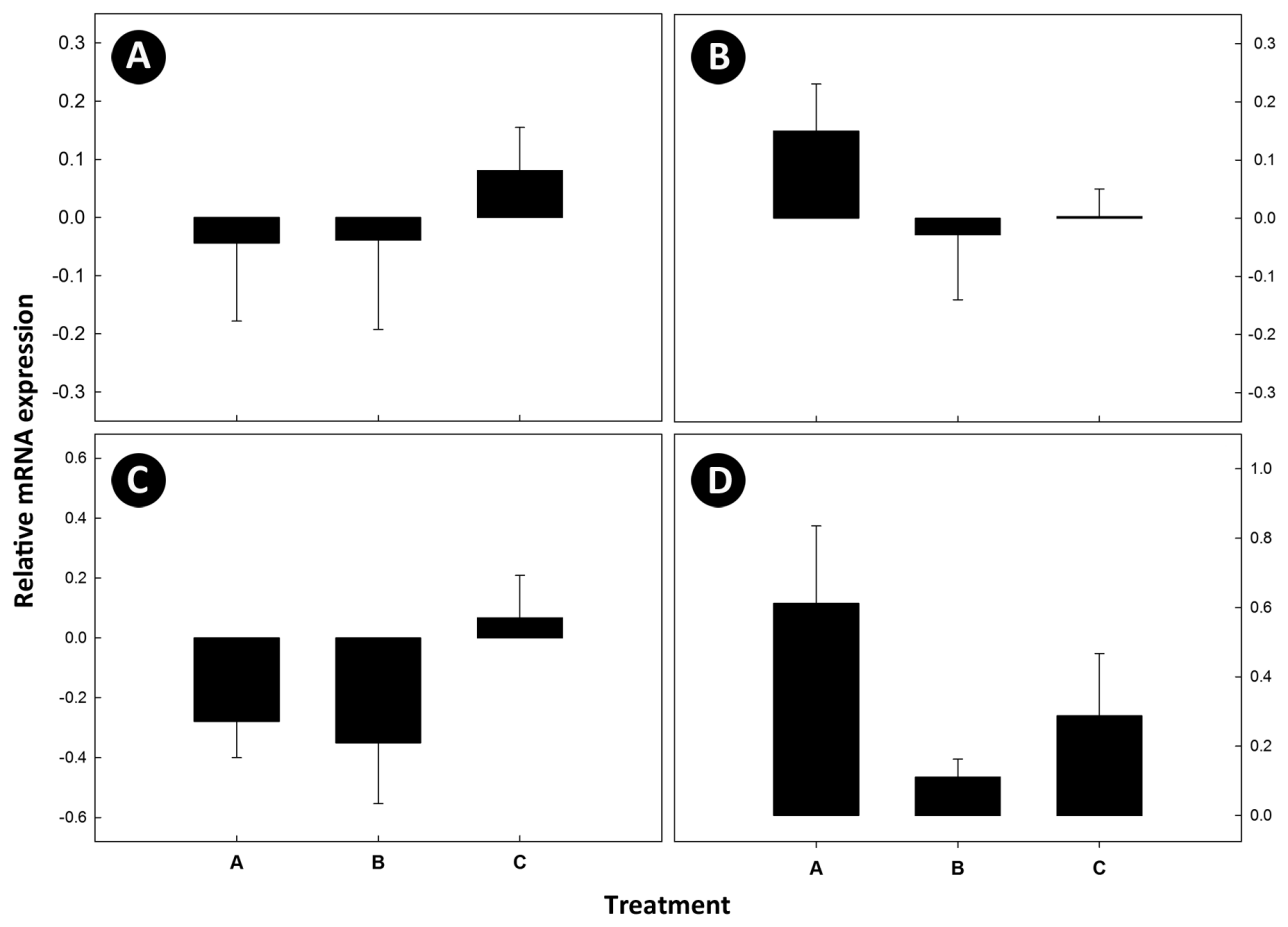
Messenger RNA expression of toll-like receptors. Relative mRNA expression of toll-like receptors (TLR) in cecal tissue where: (A) TLR2; (B) TLR4; (C) TLR5; and (D) TLR9. Treatments are: (A) *C*. *jejuni* Group A (8.8 log_10_ copy number of *C*. *jejuni* g^-1^ of cecal tissue); (B) *C*. *jejuni* Group B (6.4 log_10_ copy number of *C*. *jejuni* g^-1^ of cecal tissue); and (C) control (not inoculated with *C*. *jejuni*). Vertical lines associated with histogram bars are standard error of the means (n=6).

**Figure 3:**
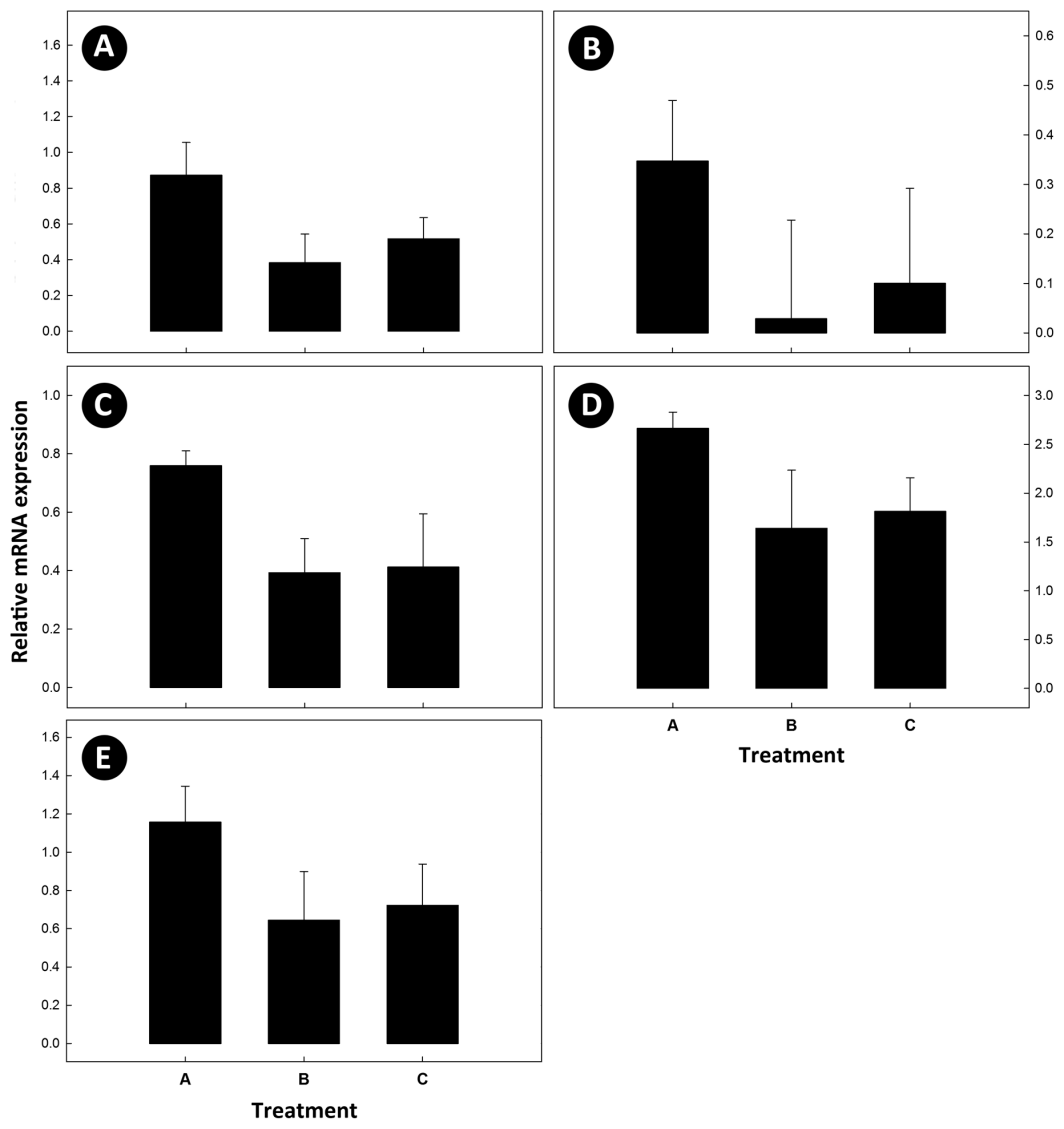
Messenger RNA expression of cytokines. Relative mRNA expression of cytokines in cecal tissue where: (A) interleukin (IL)-1β; (B) IL-6; (C) IL-10; (D) interferon-γ; and (E) tumor necrosis factor-α. Treatments are: (A) *C*. *jejuni* Group A (8.8 log_10_ copy number of *C*. *jejuni* g^-1^ of cecal tissue); (B) *C*. *jejuni* Group B (6.4 log_10_ copy number of *C*. *jejuni* g^-1^ of cecal tissue); and (C) control (not inoculated with *C*. *jejuni*). Vertical lines associated with histogram bars are standard error of the means (n=6).

No amplification of cryptdin 4 was detected, and no difference (P≥0.52) was observed in mRNA expression of the α-defensins, cryptdin 5, and cryptdin 20 among the three treatments. There also was no difference (P≥0.12) among treatments in the regulation of mRNA for toll-like receptor genes (TLR2, TLR4, TLR5, and TLR9) (Figure 2). However, a trend for increased expression of mRNA was observed in cecal tissue from *C. jejuni* Group A mice for TLR4 and TLR9. No amplification of mRNA for the Treg cytokine, TNF-β, the Th17 cytokines, IL-17A and IL-22, or the Th2 cytokines, IL-4 and IL-5 was observed. Although not significant (P≥0.056), a trend for up regulation of IL-1β, IL-6, IL-10, INF-γ, and TNF-α mRNA was observed for *C. jejuni* Group A mice (Figure 3).

### *Campylobacter jejuni* colonization was associated with a dysbiosis in the cecal microbiota

In the current study, analyses of the microbiota were limited to the mucosa-associated microbiota of the cecum. A decision was made to focus on the cecum because *C. jejuni* readily and persistently colonizes this region of the GIT in mice [29]. The total bacterial load in cecum was significantly (P<0.05) reduced in *C. jejuni* Group A mice. In contrast, no difference (P<0.05) in total number of bacteria associated with the cecal mucosa of *C. jejuni* Group B and Control mice was observed (Table 2).

**Figure 4:**
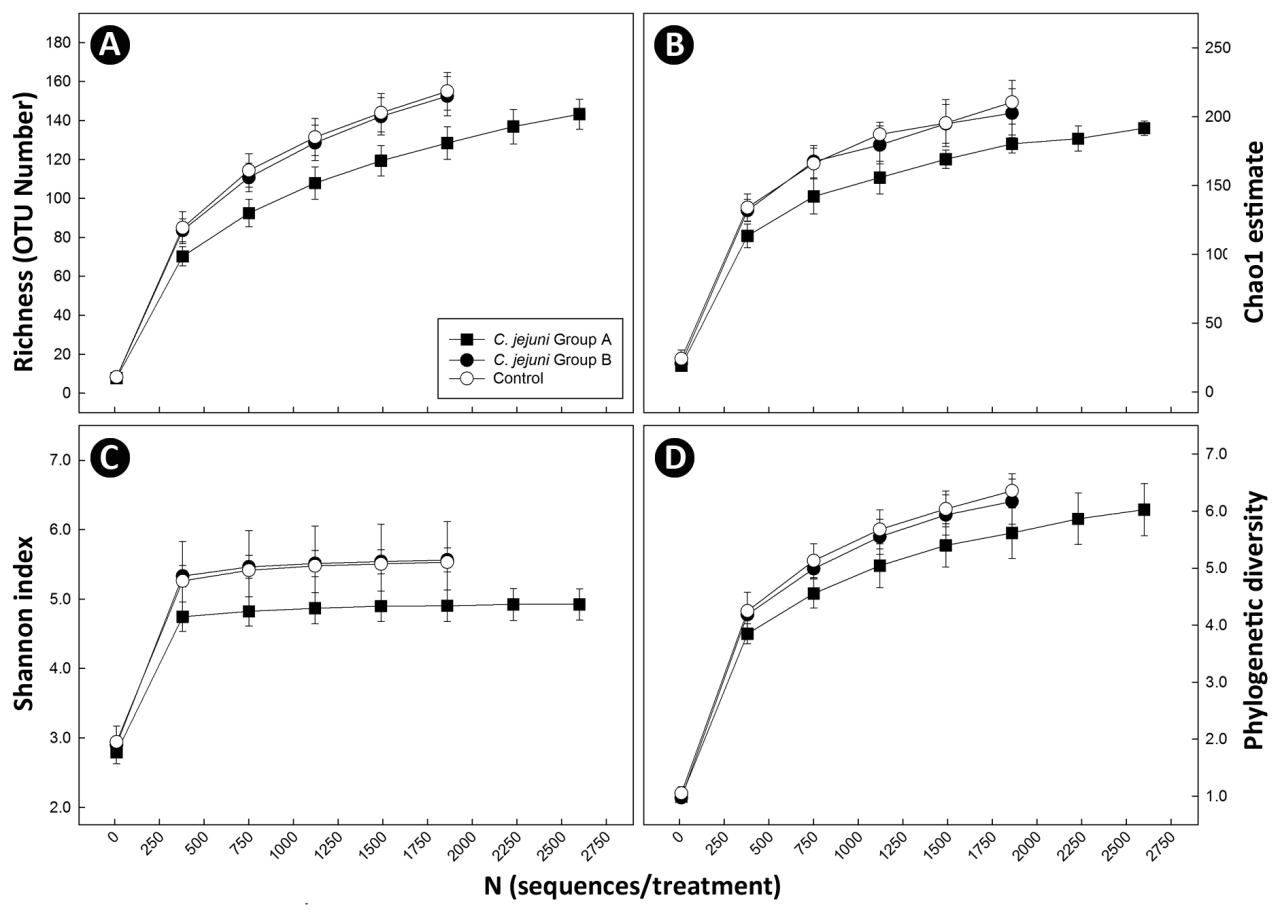
Richness and diversity of bacterial communities. (A) Richness; (B) Chao1 diversity; (C) Shannon diversity; and (D) phylogenetic diversity. Treatments are: (A) *C*. *jejuni* Group A (8.8 log_10_ copy number of *C*. *jejuni* g^-1^ of cecal tissue); (B) *C*. *jejuni* Group B (6.4 log_10_ copy number of *C*. *jejuni* g^-1^ of cecal tissue); and (C) control (not inoculated with *C*. *jejuni*). Vertical lines associated with markers are standard error of the means (n=6).

**Figure 5:**
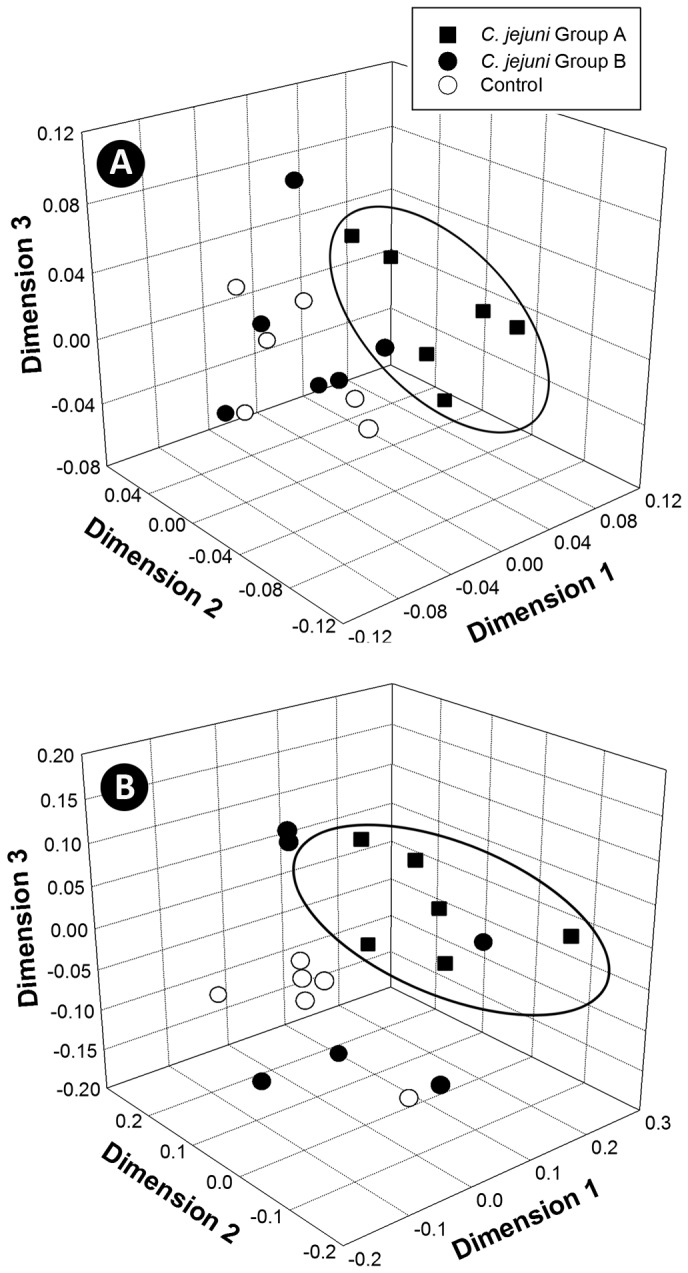
Principal coordinate cluster plots of bacterial communities. Plots depict community composition similarities based on pyrosequence analysis of: (A) weighted; and (B) unweighted datasets subsampled to an even depth. Treatments are: *C*. *jejuni* Group A (8.8 log_10_ copy number of *C*. *jejuni* g^-1^ of cecal tissue); *C*. *jejuni* Group B (6.4 log_10_ copy number of *C*. *jejuni* g^-1^ of cecal tissue); and control (not inoculated with *C*. *jejuni*). The ellipsoids show predominant clustering of bacterial communities in *C*. *jejuni* Group A mice relative to *C*. *jejuni* Group B and control mice.

**Figure 6:**
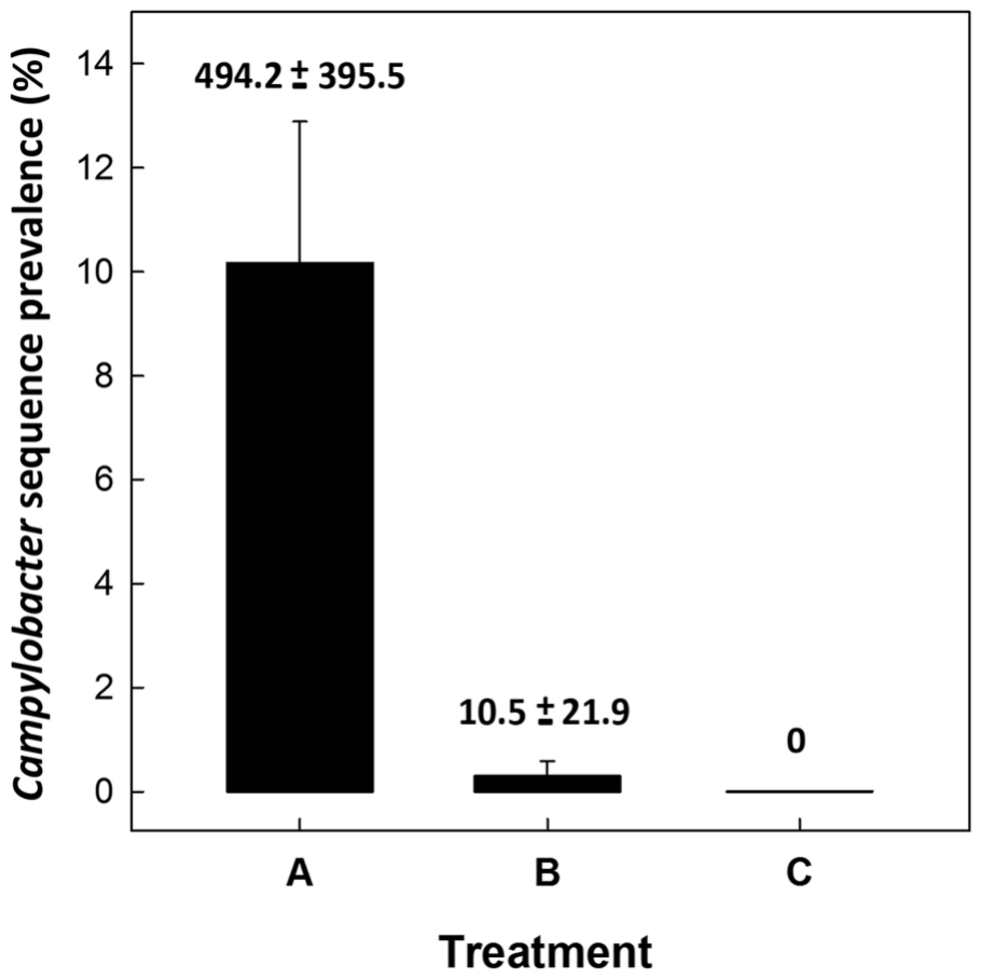
Prevalence of *Campylobacter jejuni* sequences. Prevalence of sequences (%) identified as *C*. *jejuni* by treatment. Treatments are: (A) *C*. *jejuni* Group A (8.8 log_10_ copy number of *C*. *jejuni* g^-1^ of cecal tissue); (B) *C*. *jejuni* Group B (6.4 log_10_ copy number of *C*. *jejuni* g^-1^ of cecal tissue); and (C) control (not inoculated with *C*. *jejuni*). Vertical lines associated with histogram bars are standard error of the means (n=6). Number associated with histogram bars are the mean number of sequences (± standard error of the means) that were identified as *C*. *jejuni* by treatment.

**Figure 7:**
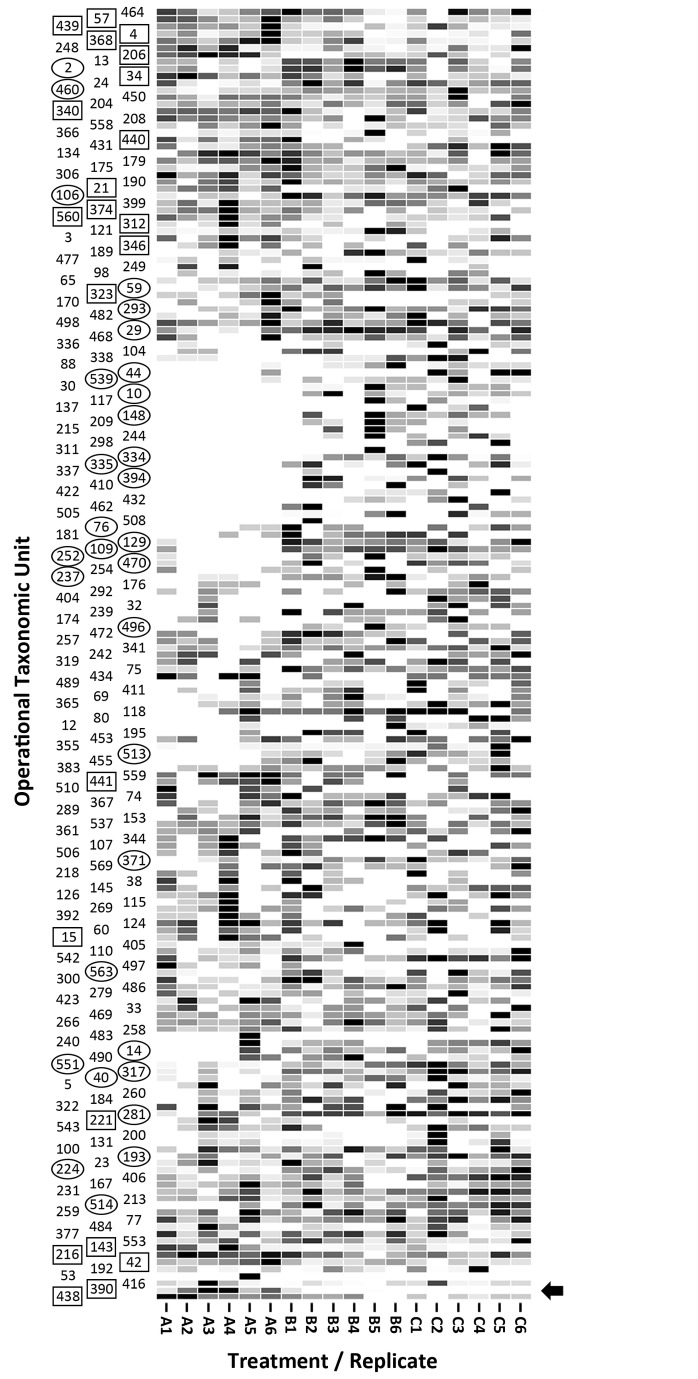
Heat map of sequence frequencies. The map shows the relative prevalence of 183 OTUs by mouse within treatment (i.e. OTUs in which ten or more sequences were observed). Treatments are: (A) *C*. *jejuni* Group A (8.8 log_10_ copy number of *C*. *jejuni* g^-1^ of cecal tissue); (B) *C*. *jejuni* Group B (6.4 log_10_ copy number of *C*. *jejuni* g^-1^ of cecal tissue); and (C) control (not inoculated with *C*. *jejuni*). The arrow indicates the OTU corresponding to *C*. *jejuni* (i.e. #390). OTU within circles represent OTU that occur conspicuously less frequently in *C*. *jejuni* Group A relative to *C*. *jejuni* Group B and Control mice. OTU within boxes represent OTU that occur conspicuously more frequently in *C*. *jejuni* Group A relative to *C*. *jejuni* Group B and Control mice. A list of taxonomic classifications by OTU is available in Table S1.

Two methods were used to characterize the mucosa-associated microbiota of the cecum. Pyrosequence analysis targeting the variable region of the 16S rRNA gene spanning the V1, V2, and V3 regions was applied. At a 97% sequence identity delineation for species [20,51], 571 OTUs were observed for the 87,492 total sequences processed (i.e. before normalization). For all animals, rarefaction curves did not asymptote (Figure 4). The richness of communities associated with the cecal mucosa of *C. jejuni* Group A mice was reduced (P=0.015) relative to *C. jejuni* Group B and Control mice (Table 4; Figure 4). A trend for decreased diversity of bacterial communities was also observed in *C. jejuni* Group A mice (Figure 4). The composition of the mucosa-associated cecal microbiota differed conspicuously between the three treatments (Figure 5), and bacterial communities in the ceca of *C. jejuni* group A mice formed distinct clades (P ≤ 0.005) from *C. jejuni* group B and Control mice (Table 5). Consistent with culture- and qPCR-based enumeration results (Tables 1-2), substantially more *C. jejuni* OTU were measured in DNA from the cecal mucosa of *C. jejuni* Group A (494.2 ± 395.5) relative to *C. jejuni* Group B (10.5 ± 21.9) mice (Figure 6). Firmicutes were the most prevalent (66.2-90.4%) group of bacteria detected. A comparison of OTU prevalence by treatment revealed a decrease in the occurrence of OTU 2, 10, 14, 29, 40, 44, 59, 76, 106, 109, 129, 148, 193, 224, 237, 252, 281, 293, 317, 334, 335, 371, 394, 460, 470, 496, 513, 514, 539, 551, and 563 in *C. jejuni* Group A relative to *C. jejuni* group B and Control mice (Figure 7; Table S1); with the exception of OTU 40, 109, 252, and 334 which was unidentified beyond the Kingdom level, all of these OTU were clostridia (*Coriobacteriaceae*, *Lachnospiraceae*, and *Ruminococcaceae*). An increase in the frequency of a number of OTU was also observed in *C. jejuni* Group A mice (4, 15, 21, 34, 42, 57, 143, 206, 216, 221, 312, 323, 340, 346, 368, 374, 390, 438, 439, 440, 441, and 560). With the exception of OTU 390 (*C. jejuni*) and 206 (unidentified), all were Firmicutes (Clostridia and Erysipelotrichi).

**Table 4:** Probability values from pairwise comparisons of bacterial richness and diversity^a^.

**Treatment group^b^**	**Richness**	**Chao1**	**Shannon**	**Phylogenetic**
Group A vs Group B	0.015	0.096	0.003	0.228
Group A vs Control	0.015	0.012	0.156	0.042
Group B vs Control	1.000	1.000	1.000	1.000

a Pairwise t-tests were conducted on data was normalized to 1916 sequences per sample.

b Group A and Group B mice were inoculated with *C. jejuni*, whereas Control mice were gavaged with buffer alone

**Table 5:** Probability values from pairwise cluster analyses of bacterial communities.

**Treatment group^a^**	**Pyrosequence^b^**		**T-RFLP**
	**(Weighted**)	**(Unweighted**)	**(T-RF Presence**)
Group A vs Group B	0.001	0.005	0.002
Group A vs Control	0.001	0.003	0.005
Group B vs Control	0.442	0.303	0.106

a Group A and Group B mice were inoculated with *C. jejuni*, whereas Control mice were gavaged with buffer alone.

b Analysis of similarity was performed on weighted and unweighted pyrosequence datasets (subsampled to an even depth).

**Figure 8:**
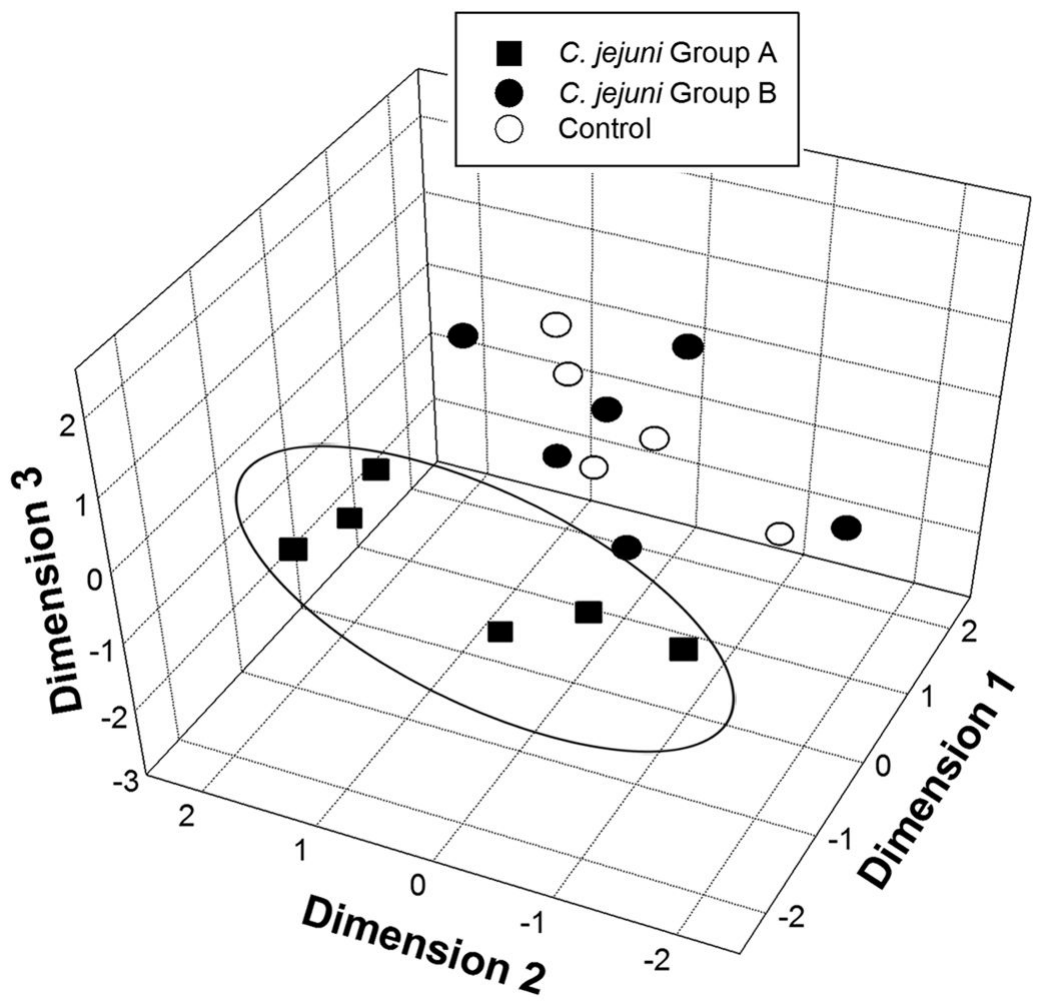
Non-metric multi-dimensional scaling plots of bacterial communities. Plots depict community terminal restriction fragment (T-RF) T-RF presence/absence of bacteria associated with mucosa within the cecum of mice. Treatments are: *C*. *jejuni* Group A (8.8 log_10_ copy number of *C*. *jejuni* g^-1^ of cecal tissue); *C*. *jejuni* Group B (6.4 log_10_ copy number of *C*. *jejuni* g^-1^ of cecal tissue); and control (not inoculated with *C*. *jejuni*). The ellipsoid shows clustering of bacterial communities in *C*. *jejuni* Group A mice relative to *C*. *jejuni* Group B and control mice.

A second method (T-RFLP fingerprint analysis) was applied to characterize bacterial communities associated with the mucosa of mice ceca. Similarly to pyrosequence-based analysis, T-RFLP analysis indicated that the cecal microbiota of *C. jejuni* Group A mice clustered separately (P≤0.005) from both *C. jejuni* Group B and Control mice (Table 5; Figure 8).

## Discussion

The role of the intestinal microbiota on *C. jejuni* colonization is poorly understood, particularly in asymptomatic *C. jejuni* carrier animals. We chose to use mice as a model given *C. jejuni* does not naturally colonize the intestine of mice, and hence it is not adapted to the murine GIT ecosystem [14]. However, *C. jejuni* readily colonizes the intestine of mice with a simplified or altered microbiota [6,26,52,53], and large doses of *C. jejuni* are typically required to experimentally colonize mice possessing a naturally-acquired microbiota [29,50,54]. Both of these observations suggest that *C. jejuni* may affect the composition of the microbiota in order to successfully colonize the GIT of asymptomatic mammals.

To study the *C. jejuni*-microbiota interaction, we examined the microbiota associated with the cecum, specifically the mucosa-associated microbiota, because the cecum is a preferred site of colonization by *C. jejuni* in mice [29], as well as in poultry [55]. Substantial densities of *C. jejuni* cells were observed in association with the mucosa of the cecum in all the inoculated mice included in the study. In contrast, *C. jejuni* was not detected or detected at low cell densities in the stomach, jejunum and ileum. The bacterium was also consistently detected in the distal colon, but at lower densities than in the cecum. We specifically targeted the mucosa-associated microbiota because luminal contents (ingesta) are not necessarily representative of the localized microbiota, and at any particular location within the intestinal lumen, ingesta carries microorganisms from the proximal regions of the GIT. Thus, examination of the luminal microbiota may provide an inaccurate representation of the localized microbiota. Additionally, bacteria within ingesta in the intestinal lumen encounter a different micro-environment than bacteria closely associated with the mucosal surface which are influenced by host factors to a much greater degree [56].

We observed two distinct groups of mice based on the density of *C. jejuni* cells shed in feces and associated with the cecal and colonic mucosa; one group of mice was colonized by *C. jejuni* at a much higher density than the second group. *Campylobacter jejuni* colonization in mice varies amongst individuals, and *C. jejuni* strains exhibit inconsistent and highly variable colonization ability [30,57]. For example, ^≈^10^5^ fold variation in *C. jejuni* NCTC 11168 densities was observed within cecal ingesta amongst C57BL/6J IL-10 deficient mice 7 days p.i. [30]. Similarly, we frequently observe variable colonization of C57BL/6J mice between and within *C. jejuni* strains, including NCTC 11168 (unpublished).

All methods to characterize the intestinal microbiota possess strengths as well as weaknesses [58]. Thus, we applied two methods to characterize the mucosa-associated microbiota within the cecum of mice (pyrosequence and T-RFLP). Sequence-based analysis of microbial community composition showed that bacterial diversity was high in all samples, and rarefaction curves did not asymptote indicating that not all the taxa present in the community were represented. However, microbial community rarefaction curves, particularly for diverse communities within the GIT typically do not saturate even at high levels of coverage provided by pyrosequencing [59,60]. Sequence based-analysis grouped the cecal mucosa-associated microbiota of mice colonized at a high cell density of *C. jejuni* cells (Group A) as a distinct cluster. In contrast, there was no difference in the composition of bacterial community between mice colonized by *C. jejuni* at the lower density (Group B) and *C. jejuni*-free (Control) animals. Thus, the composition of the cecal microbiota of *C. jejuni* Group A mice was distinct from the other two treatments. The particular composition of bacterial communities might be responsible for differential colonization of *C. jejuni* in dissimilar animal species, but the high degree of inter-individual variability typically observed within a particular species is problematic [61]. Our data contrasts with an earlier report that concluded that *C. jejuni* colonization of the mouse intestine did not significantly affect bacterial load or the composition of the enteric microbiota within the colons of IL-10 deficient mice [14]; however, *C. jejuni* colonization density was not specifically considered, and characterization of the microbiota was limited to enumeration of a limited number of bacterial groups using fluorescence *in situ* hybridization. Although not significant, they did observe a trend for reduced bacterial load [14]. We observed that the mucosa-associated bacterial community was dominated by Firmicutes, and a number of Clostridia OTU (primarily *Coriobacteriaceae*, *Lachnospiraceae*, and *Ruminococcaceae*) were either less or more frequently observed in mice colonized by *C. jejuni* at high densities. The role of these bacteria in colonization resistance warrants further investigation.

To confirm the pyrosequence-based community composition results, T-RFLP analysis was applied as an alternate method (e.g. to address the possibility that the high frequency measurement of *C. jejuni* sequences in *C. jejuni* Group A mice skewed richness, diversity, and composition metrics). Although the T-RFLP method is not capable of identifying constituents of the community, it is a method that generates highly reproducible community fingerprints that facilitates rapid and cost-effective comparative characterization of communities [62,63]. In this regard, Pilloni et al. [64] observed that T-RFLP analysis was able to recover the same amplicon pools from environmental samples, and yielded highly comparable overall microbial community patterns to pyrosequencing, but may underestimate diversity. The application of the T-RFLP method confirmed that the composition of the mucosa-associated microbiota in ceca of mice colonized by a high density of *C. jejuni* cells (Group A) was distinct. The findings of our study clearly demonstrate that high density colonization by *C. jejuni* was associated with a dysbiosis in the cecal microbiota.

The dysbiosis that we observed may have been incited by *C. jejuni*, or may have resulted from unknown event(s) that caused a dysbiosis thereby permitting *C. jejuni* to colonize the cecal mucosa at high densities. Host factors, including differential immune competence can influence the composition of the microbiota within individual animals [65,66]. We exercised care to ensure that mice were treated identically and randomly assigned to treatments. Furthermore, the composition of the microbiota of all Control mice grouped together. Collectively, this suggests that *C. jejuni* directly affected the cecal microbiota. Inflammation incited by other enteric pathogens has been documented to influence the composition of the intestinal microbiota [14,17,67]. However, *C. jejuni* did not incite prominent inflammation in the current study. This was evident by the absence of clinical signs, and gross and microscopic indications of intestinal inflammation. In addition, we did not detect a statistically significant difference in growth rate over the experimental period between *C. jejuni*-infected and Control mice. Furthermore, we did not observe an increase in the prevalence of *Enterobacteriaceae* bacteria which is often observed in association with inflamed intestines [6,14]. Similarly to previous reports [14,27,28,29,30], we observed that *C. jejuni* did not incite prominent intestinal inflammation in mice (e.g. histopathologic changes). Total histopathologic scores were ≤4.2 in the current study, whereas total scores exceeding 10 (i.e. marked changes) are recorded in C57/6J mice with acute enteritis [33]. Consistent with this observation, non-significant differences were observed among treatments in the expression of mRNA for α-defensins, toll-like receptors, or cytokines. Of note, increases in expression of cytokine mRNA (e.g. INF-ɣ, TNF-α, IL-2) exceeding five-fold are typically observed in mice with acute enteritis [33,68,69]. Although α-defensins (termed cryptdins in mice) are primarily expressed in Paneth cells concentrated in the small intestine, Paneth cells can also be present in cecum and colon [70]. Expression of α-defensins can modulate intestinal inflammation and tissue injury, as well as the microbiota [71,72,73]. Our data showed that Cryptdin 4, 5, or 20 are not induced by *C. jejuni*, and that they do not play a role in facilitating cecal colonization in mice. Although non-significant, we observed trends of differential regulation of toll-like receptor and cytokine genes in mice colonized at a high cell density by *C. jejuni* (Group A) relative to mice in other treatments. The toll-like receptors, TLR4 and TLR9 are important molecules for the recognition of lipopolysaccharide moieties of Gram negative bacteria and unmethylated CpG rich regions of bacterial DNA respectively, and the modest increase in gene expression observed in the current study is consistent with other research that identified enhanced TLR signalling with the induction of immunopathology due to *C. jejuni*-infection in knockout mice [53]. Furthermore, it is known that *C. jejuni* modulates the expression of proinflammatory cytokines in a variety of murine models consistent with our findings [6,53,74,75,76]. *Salmonella enterica* has been shown to exploit the inflammatory response to compete with the enteric microbiota [16,17,77,78,79]. Our data suggests that *C. jejuni* incites a low-grade inflammation response as a colonization strategy in asymptomatic hosts, but the bacterium is unable to do so in all individuals due to unknown factors. Besides exploiting the host, it is also possible that *C. jejuni* affects the microbiota or that constituents of the microbiota affect *C. jejuni* independent of the host, as has been observed for other pathogenic bacteria [80,81,82,83]. In conclusion, we observed that high density colonization of the cecum by *C. jejuni* was associated with a dysbiosis in the cecal microbiota independent of prominent inflammation. Although our research identifies a unique aspect by which *C. jejuni* impacts on the host and the intestinal microbiota, future research to elucidate the mechanisms is warranted.

## Supporting Information

Table S1 **Identities of operational taxonomic units (OTUs).** See Figure 6 for the relative frequency of individual OTUs by sample within treatments (i.e. OTUs for which ten sequences or more were observed).
(DOCX)

## References

[1] Allos BM (2001) *Campylobacter* *jejuni* Infections: update on emerging issues and trends. Clin Infect Dis 32: 1201-1206. doi:10.1086/319760. PubMed: 11283810. 11283810 10.1086/319760

[2] Newell DG, Fearnley C (2003) Sources of *Campylobacter* colonization in broiler chickens. Appl Environ Microbiol 69: 4343-4351. doi:10.1128/AEM.69.8.4343-4351.2003. PubMed: 12902214. 12902214 10.1128/AEM.69.8.4343-4351.2003 PMC169125

[3] Young KT, Davis LM, Dirita VJ (2007) *Campylobacter* *jejuni*: molecular biology and pathogenesis. Nat Rev Microbiol 5: 665-679. doi:10.1038/nrmicro1718. PubMed: 17703225. 17703225 10.1038/nrmicro1718

[4] Cone LA, Dreisbach PB, Hirschberg J, Shekar C, Dreisbach LP et al. (2003) Cellulitis and septic arthritis caused by *Campylobacter* *fetus* and *Campylobacter* *jejuni*: report of 2 cases and review of the literature. J Clin Rheumatol 9: 362-369. doi:10.1097/01.rhu.0000090261.11345.87. PubMed: 17043445. 17043445 10.1097/01.rhu.0000090261.11345.87

[5] Kalischuk LD, Buret AG (2010) A role for *Campylobacter* *jejuni*-induced enteritis in inflammatory bowel disease? Am J Physiol Gastrointest Liver Physiol 298: G1-G9. doi:10.1152/ajpgi.00193.2009. PubMed: 19875702. 19875702 10.1152/ajpgi.00193.2009

[6] Haag LM, Fischer A, Otto B, Plickert R, Kühl AA et al. (2012) Intestinal microbiota shifts towards elevated commensal *Escherichia* *coli* loads abrogate colonization resistance against *Campylobacter* *jejuni* in mice. PLOS ONE 7: e35988. doi:10.1371/journal.pone.0035988. PubMed: 22563475. 22563475 10.1371/journal.pone.0035988 PMC3341396

[7] Davis L, DiRita V (2008) Experimental chick colonization by *Campylobacter* *jejuni* . Curr Protoc Microbiol Chapter 8: Unit 8A 3. PubMed: 19016444 10.1002/9780471729259.mc08a03s11 PMC5147583 19016444

[8] Kwan PS, Barrigas M, Bolton FJ, French NP, Gowland P et al. (2008) Molecular epidemiology of *Campylobacter* *jejuni* populations in dairy cattle, wildlife, and the environment in a farmland area. Appl Environ Microbiol 74: 5130-5138. doi:10.1128/AEM.02198-07. PubMed: 18586964. 18586964 10.1128/AEM.02198-07 PMC2519278

[9] Coker AO, Isokpehi RD, Thomas BN, Amisu KO, Obi CL (2002) Human campylobacteriosis in developing countries. Emerg Infect Dis 8: 237-244. doi:10.3201/eid0803.010233. PubMed: 11927019. 11927019 10.3201/eid0803.010233 PMC2732465

[10] Calva JJ, Ruiz-Palacios GM, Lopez-Vidal AB, Ramos A, Bojalil R (1988) Cohort study of intestinal infection with campylobacter in Mexican children. Lancet 1: 503-506. PubMed: 2893920. 2893920 10.1016/s0140-6736(88)91297-4

[11] Figueroa G, Galeno H, Troncoso M, Toledo S, Soto V (1989) Prospective study of *Campylobacter* *jejuni* infection in Chilean infants evaluated by culture and serology. J Clin Microbiol 27: 1040-1044. PubMed: 2473090. 2473090 10.1128/jcm.27.5.1040-1044.1989 PMC267479

[12] Bäckhed F, Ley RE, Sonnenburg JL, Peterson DA, Gordon JI (2005) Host-bacterial mutualism in the human intestine. Science 307: 1915-1920. doi:10.1126/science.1104816. PubMed: 15790844. 15790844 10.1126/science.1104816

[13] Endt K, Stecher B, Chaffron S, Slack E, Tchitchek N et al. (2010) The microbiota mediates pathogen clearance from the gut lumen after non-typhoidal *Salmonella* diarrhea. PLOS Pathog 6: e1001097 PubMed: 20844578. 20844578 10.1371/journal.ppat.1001097 PMC2936549

[14] Lupp C, Robertson ML, Wickham ME, Sekirov I, Champion OL et al. (2007) Host-mediated inflammation disrupts the intestinal microbiota and promotes the overgrowth of Enterobacteriaceae. Cell Host Microbe 2: 204. doi:10.1016/j.chom.2007.08.002. PubMed: 18030708. 18030708 10.1016/j.chom.2007.08.002

[15] Stecher B, Hardt WD (2008) The role of microbiota in infectious disease. Trends Microbiol 16: 107-114. doi:10.1016/j.tim.2007.12.008. PubMed: 18280160. 18280160 10.1016/j.tim.2007.12.008

[16] Winter SE, Bäumler AJ (2011) A breathtaking feat: to compete with the gut microbiota, *Salmonella* drives its host to provide a respiratory electron acceptor. Gut Microbes 2: 58-60. PubMed: 21637020. 21637020 10.4161/gmic.2.1.14911 PMC3225798

[17] Thiennimitr P, Winter SE, Bäumler AJ (2012) *Salmonella*, the host and its microbiota. Curr Opin Microbiol 15: 108-114. doi:10.1016/j.mib.2011.10.002. PubMed: 22030447. 22030447 10.1016/j.mib.2011.10.002 PMC3265626

[18] Barthel M, Hapfelmeier S, Quintanilla-Martínez L, Kremer M, Rohde M et al. (2003) Pretreatment of mice with streptomycin provides a *Salmonella* *enterica* serovar Typhimurium colitis model that allows analysis of both pathogen and host. Infect Immun 71: 2839-2858. doi:10.1128/IAI.71.5.2839-2858.2003. PubMed: 12704158. 12704158 10.1128/IAI.71.5.2839-2858.2003 PMC153285

[19] Croswell A, Amir E, Teggatz P, Barman M, Salzman NH (2009) Prolonged impact of antibiotics on intestinal microbial ecology and susceptibility to enteric *Salmonella* infection. Infect Immun 77: 2741-2753. doi:10.1128/IAI.00006-09. PubMed: 19380465. 19380465 10.1128/IAI.00006-09 PMC2708550

[20] Bailey MT, Dowd SE, Parry NM, Galley JD, Schauer DB et al. (2010) Stressor exposure disrupts commensal microbial populations in the intestines and leads to increased colonization by *Citrobacter* *rodentium* . Infect Immun 78: 1509-1519. doi:10.1128/IAI.00862-09. PubMed: 20145094. 20145094 10.1128/IAI.00862-09 PMC2849416

[21] Britton RA, Young VB (2012) Interaction between the intestinal microbiota and host in *Clostridium* *difficile* colonization resistance. Trends Microbiol 20: 313-319. doi:10.1016/j.tim.2012.04.001. PubMed: 22595318. 22595318 10.1016/j.tim.2012.04.001 PMC3408078

[22] Stecher B, Hardt WD (2011) Mechanisms controlling pathogen colonization of the gut. Curr Opin Microbiol 14: 82-91. doi:10.1016/j.mib.2010.10.003. PubMed: 21036098. 21036098 10.1016/j.mib.2010.10.003

[23] Inglis GD, Kalischuk LD, Busz HW, Kastelic JP (2005) Colonization of cattle intestines by *Campylobacter* *jejuni* and *Campylobacter* *lanienae* . Appl Environ Microbiol 71: 5145-5153. doi:10.1128/AEM.71.9.5145-5153.2005. PubMed: 16151098. 16151098 10.1128/AEM.71.9.5145-5153.2005 PMC1214653

[24] Hermans D, Van Deun K, Martel A, Van Immerseel F, Messens W et al. (2011) Colonization factors of *Campylobacter* *jejuni* in the chicken gut. Vet Res 42: 82. doi:10.1186/1297-9716-42-82. PubMed: 21714866. 21714866 10.1186/1297-9716-42-82 PMC3156733

[25] Fox JG, Zanotti S, Jordan HV, Murphy JC (1986) Colonization of Syrian hamsters with streptomycin resistant *Campylobacter* *jejuni* . Lab Anim Sci 36: 28-31. PubMed: 3959531. 3959531

[26] Jesudason MV, Hentges DJ, Pongpech P (1989) Colonization of mice by *Campylobacter* *jejuni* . Infect Immun 57: 2279-2282. PubMed: 2744846. 2744846 10.1128/iai.57.8.2279-2282.1989 PMC313442

[27] Rinella ES, Eversley CD, Carroll IM, Andrus JM, Threadgill DW et al. (2006) Human epithelial-specific response to pathogenic *Campylobacter* *jejuni* . FEMS Microbiol Lett 262: 236-243. doi:10.1111/j.1574-6968.2006.00396.x. PubMed: 16923081. 16923081 10.1111/j.1574-6968.2006.00396.x

[28] Dorrell N, Wren BW (2007) The second century of *Campylobacter* *research*: recent advances, new opportunities and old problems. Curr Opin Infect Dis 20: 514-518. doi:10.1097/QCO.0b013e3282a56b15. PubMed: 17762786. 17762786 10.1097/QCO.0b013e3282a56b15

[29] Mansfield LS, Bell JA, Wilson DL, Murphy AJ, Elsheikha HM et al. (2007) C57BL/6 and congenic interleukin-10-deficient mice can serve as models of *Campylobacter* *jejuni* colonization and enteritis. Infect Immun 75: 1099-1115. doi:10.1128/IAI.00833-06. PubMed: 17130251. 17130251 10.1128/IAI.00833-06 PMC1828563

[30] Wilson DL, Rathinam VA, Qi W, Wick LM, Landgraf J et al. (2010) Genetic diversity in *Campylobacter* *jejuni* is associated with differential colonization of broiler chickens and C57BL/6J IL10-deficient mice. Microbiology 156: 2046-2057. doi:10.1099/mic.0.035717-0. PubMed: 20360176. 20360176 10.1099/mic.0.035717-0 PMC3068676

[31] Taboada EN, Ross SL, Mutschall SK, Mackinnon JM, Roberts MJ et al. (2012) Development and validation of a comparative genomic fingerprinting method for high-resolution genotyping of *Campylobacter* *jejuni* . J Clin Microbiol 50: 788-797. doi:10.1128/JCM.00669-11. PubMed: 22170908. 22170908 10.1128/JCM.00669-11 PMC3295178

[32] Inglis GD, Kalischuk LD (2003) Use of PCR for direct detection of *Campylobacter* species in bovine feces. Appl Environ Microbiol 69: 3435-3447. doi:10.1128/AEM.69.6.3435-3447.2003. PubMed: 12788747. 12788747 10.1128/AEM.69.6.3435-3447.2003 PMC161499

[33] Costa E, Uwiera RR, Kastelic JP, Selinger LB, Inglis GD (2011) Non-therapeutic administration of a model antimicrobial growth promoter modulates intestinal immune responses. Gut Pathog 3: 14. doi:10.1186/1757-4749-3-14. PubMed: 21943280. 21943280 10.1186/1757-4749-3-14 PMC3195107

[34] Inglis GD, Kastelic JP, Uwiera RR (2010) Catheterization of intestinal loops in ruminants does not adversely affect loop function. Comp Med 60: 469-478. PubMed: 21262134. 21262134 PMC3002107

[35] Vandesompele J, De Preter K, Pattyn F, Poppe B, Van Roy N et al. (2002) Accurate normalization of real-time quantitative RT-PCR data by geometric averaging of multiple internal control genes. Genome Biol 3: RESEARCH0034 PubMed: 12184808. 12184808 10.1186/gb-2002-3-7-research0034 PMC126239

[36] Reiner SL, Zheng S, Corry DB, Locksley RM (1993) Constructing polycompetitor cDNAs for quantitative PCR. J Immunol Methods 165: 37-46. doi:10.1016/0022-1759(93)90104-F. PubMed: 8409467. 8409467 10.1016/0022-1759(93)90104-f

[37] Hellemans J, Mortier G, De Paepe A, Speleman F, Vandesompele J (2007) qBase relative quantification framework and software for management and automated analysis of real-time quantitative PCR data. Genome Biol 8: R19. doi:10.1186/gb-2007-8-2-r19. PubMed: 17291332. 17291332 10.1186/gb-2007-8-2-r19 PMC1852402

[38] Inglis GD, Kalischuk LD (2004) Direct quantification of *Campylobacter* *jejuni* and *Campylobacter* *lanienae* in feces of cattle by real-time quantitative PCR. Appl Environ Microbiol 70: 2296-2306. doi:10.1128/AEM.70.4.2296-2306.2004. PubMed: 15066825. 15066825 10.1128/AEM.70.4.2296-2306.2004 PMC383034

[39] Walter J, Tannock GW, Tilsala-Timisjarvi A, Rodtong S, Loach DM et al. (2000) Detection and identification of gastrointestinal *Lactobacillus* species by using denaturing gradient gel electrophoresis and species-specific PCR primers. Appl Environ Microbiol 66: 297-303. doi:10.1128/AEM.66.1.297-303.2000. PubMed: 10618239. 10618239 10.1128/aem.66.1.297-303.2000 PMC91821

[40] Dowd SE, Wolcott RD, Sun Y, McKeehan T, Smith E et al. (2008) Polymicrobial nature of chronic diabetic foot ulcer biofilm infections determined using bacterial tag encoded FLX amplicon pyrosequencing (bTEFAP). PLOS ONE 3: e3326. doi:10.1371/journal.pone.0003326. PubMed: 18833331. 18833331 10.1371/journal.pone.0003326 PMC2556099

[41] Ishak HD, Plowes R, Sen R, Kellner K, Meyer E et al. (2011) Bacterial diversity in *Solenopsis* *invicta* and *Solenopsis* *geminata* ant colonies characterized by 16S amplicon 454 pyrosequencing. Microb Ecol 61: 821-831. doi:10.1007/s00248-010-9793-4. PubMed: 21243351. 21243351 10.1007/s00248-010-9793-4

[42] Dowd SE, Callaway TR, Wolcott RD, Sun Y, McKeehan T et al. (2008) Evaluation of the bacterial diversity in the feces of cattle using 16S rDNA bacterial tag-encoded FLX amplicon pyrosequencing (bTEFAP). BMC Microbiol 8: 125. doi:10.1186/1471-2180-8-125. PubMed: 18652685. 18652685 10.1186/1471-2180-8-125 PMC2515157

[43] Schloss PD, Westcott SL, Ryabin T, Hall JR, Hartmann M et al. (2009) Introducing mothur: open-source, platform-independent, community-supported software for describing and comparing microbial communities. Appl Environ Microbiol 75: 7537-7541. doi:10.1128/AEM.01541-09. PubMed: 19801464. 19801464 10.1128/AEM.01541-09 PMC2786419

[44] Needleman SB, Wunsch CD (1970) A general method applicable to the search for similarities in the amino acid sequence of two proteins. J Mol Biol 48: 443-453. doi:10.1016/0022-2836(70)90057-4. PubMed: 5420325. 5420325 10.1016/0022-2836(70)90057-4

[45] Edgar RC, Haas BJ, Clemente JC, Quince C, Knight R (2011) UCHIME improves sensitivity and speed of chimera detection. Bioinformatics 27: 2194-2200. doi:10.1093/bioinformatics/btr381. PubMed: 21700674. 21700674 10.1093/bioinformatics/btr381 PMC3150044

[46] Caporaso JG, Kuczynski J, Stombaugh J, Bittinger K, Bushman FD et al. (2010) QIIME allows analysis of high-throughput community sequencing data. Nat Methods 7: 335-336. doi:10.1038/nmeth.f.303. PubMed: 20383131. 20383131 10.1038/nmeth.f.303 PMC3156573

[47] Oksanen J, Blanchet FG, Kindt R, Legendre P, Minchin PR et al. (2011). Vegan: community ecology package version 2.0-2. http://tomato.biol.trinity.edu/programs/index.php/Vegan Accessed 2013 July 9.

[48] Costa E, Puhl NJ, Selinger LB, Inglis GD (2009) Characterization of mucosa-associated bacterial communities of the mouse intestine by terminal restriction fragment length polymorphism: utility of sampling strategies and methods to reduce single-stranded DNA artifacts. J Microbiol Methods 78: 175-180. doi:10.1016/j.mimet.2009.05.011. PubMed: 19463863. 19463863 10.1016/j.mimet.2009.05.011

[49] Lane DJ (1991) 16S/23S rRNA sequencing. In: Stackebrandt E Goodfellow M Nucleic Acid Techniques in Bacterial Systematics. New York, NY.: John Wiley & Sons pp. 115–175.

[50] Bell JA, St Charles JL, Murphy AJ, Rathinam VA, Plovanich-Jones AE et al. (2009) Multiple factors interact to produce responses resembling spectrum of human disease in *Campylobacter* *jejuni* infected C57BL/6 IL-10-/- mice. BMC Microbiol 9: 57. doi:10.1186/1471-2180-9-57. PubMed: 19296832. 19296832 10.1186/1471-2180-9-57 PMC2669091

[51] Zaura E, Keijser BJ, Huse SM, Crielaard W (2009) Defining the healthy "core microbiome" of oral microbial communities. BMC Microbiol 9: 259. doi:10.1186/1471-2180-9-259. PubMed: 20003481. 20003481 10.1186/1471-2180-9-259 PMC2805672

[52] Chang C, Miller JF (2006) *Campylobacter* *jejuni* colonization of mice with limited enteric flora. Infect Immun 74: 5261-5271. doi:10.1128/IAI.01094-05. PubMed: 16926420. 16926420 10.1128/IAI.01094-05 PMC1594848

[53] Bereswill S, Fischer A, Plickert R, Haag LM, Otto B et al. (2011) Novel murine infection models provide deep insights into the "menage a trois" of *Campylobacter* *jejuni*, microbiota and host innate immunity. PLOS ONE 6: e20953. doi:10.1371/journal.pone.0020953. PubMed: 21698299. 21698299 10.1371/journal.pone.0020953 PMC3115961

[54] Mansfield LS, Patterson JS, Fierro BR, Murphy AJ, Rathinam VA et al. (2008) Genetic background of IL-10(-/-) mice alters host-pathogen interactions with *Campylobacter* *jejuni* and influences disease phenotype. Microb Pathog 45: 241-257. doi:10.1016/j.micpath.2008.05.010. PubMed: 18586081. 18586081 10.1016/j.micpath.2008.05.010 PMC4148907

[55] Li X, Swaggerty CL, Kogut MH, Chiang HI, Wang Y et al. (2010) Gene expression profiling of the local cecal response of genetic chicken lines that differ in their susceptibility to *Campylobacter* *jejuni* colonization. PLOS ONE 5: e11827. doi:10.1371/journal.pone.0011827. PubMed: 20676366. 20676366 10.1371/journal.pone.0011827 PMC2911375

[56] Zoetendal EG, von Wright A, Vilpponen-Salmela T, Ben-Amor K, Akkermans AD et al. (2002) Mucosa-associated bacteria in the human gastrointestinal tract are uniformly distributed along the colon and differ from the community recovered from feces. Appl Environ Microbiol 68: 3401-3407. doi:10.1128/AEM.68.7.3401-3407.2002. PubMed: 12089021. 12089021 10.1128/AEM.68.7.3401-3407.2002 PMC126800

[57] Bell JA, Jerome JP, Plovanich-Jones AE, Smith EJ, Gettings JR et al. (2013) Outcome of infection of C57BL/6 IL-10(-/-) mice with *Campylobacter* *jejuni* strains is correlated with genome content of open reading frames up- and down-regulated *in* *vivo* . Microb Pathog 54: 1-19. doi:10.1016/j.micpath.2012.08.001. PubMed: 22960579. 22960579 10.1016/j.micpath.2012.08.001 PMC4118490

[58] Inglis GD, Thomas MC, Thomas DK, Kalmokoff ML, Brooks SPJ et al. (2012) Methods to measure intestinal bacteria: a review. J AOAC Int 95: 5-23. doi:10.5740/jaoacint.SGE_Inglis. PubMed: 22468337. 22468337 10.5740/jaoacint.sge_inglis

[59] Dethlefsen L, Huse S, Sogin ML, Relman DA (2008) The pervasive effects of an antibiotic on the human gut microbiota, as revealed by deep 16S rRNA sequencing. PLOS Biol 6: e280. doi:10.1371/journal.pbio.0060280. PubMed: 19018661. 19018661 10.1371/journal.pbio.0060280 PMC2586385

[60] Turnbaugh PJ, Quince C, Faith JJ, McHardy AC, Yatsunenko T et al. (2010) Organismal, genetic, and transcriptional variation in the deeply sequenced gut microbiomes of identical twins. Proc Natl Acad Sci U S A 107: 7503-7508. doi:10.1073/pnas.1002355107. PubMed: 20363958. 20363958 10.1073/pnas.1002355107 PMC2867707

[61] Spor A, Koren O, Ley R (2011) Unravelling the effects of the environment and host genotype on the gut microbiome. Nat Rev Microbiol 9: 279-290. doi:10.1038/nrmicro2540. PubMed: 21407244. 21407244 10.1038/nrmicro2540

[62] Camarinha-Silva A, Wos-Oxley ML, Jáuregui R, Becker K, Pieper DH (2012) Validating T-RFLP as a sensitive and high-throughput approach to assess bacterial diversity patterns in human anterior nares. FEMS Microbiol Ecol 79: 98-108. doi:10.1111/j.1574-6941.2011.01197.x. PubMed: 22066869. 22066869 10.1111/j.1574-6941.2011.01197.x

[63] Ruan Q, Dutta D, Schwalbach MS, Steele JA, Fuhrman JA et al. (2006) Local similarity analysis reveals unique associations among marine bacterioplankton species and environmental factors. Bioinformatics 22: 2532-2538. doi:10.1093/bioinformatics/btl417. PubMed: 16882654. 16882654 10.1093/bioinformatics/btl417

[64] Pilloni G, Granitsiotis MS, Engel M, Lueders T (2012) Testing the limits of 454 pyrotag sequencing: reproducibility, quantitative assessment and comparison to T-RFLP Fingerprinting of aquifer microbes. PLOS ONE 7: e40467. doi:10.1371/journal.pone.0040467. PubMed: 22808168. 22808168 10.1371/journal.pone.0040467 PMC3395703

[65] Benson AK, Kelly SA, Legge R, Ma F, Low SJ et al. (2010) Individuality in gut microbiota composition is a complex polygenic trait shaped by multiple environmental and host genetic factors. Proc Natl Acad Sci U S A 107: 18933-18938. doi:10.1073/pnas.1007028107. PubMed: 20937875. 20937875 10.1073/pnas.1007028107 PMC2973891

[66] Hooper LV, Macpherson AJ (2010) Immune adaptations that maintain homeostasis with the intestinal microbiota. Nat Rev Immunol 10: 159-169. doi:10.1038/nri2710. PubMed: 20182457. 20182457 10.1038/nri2710

[67] Hoffmann C, Hill DA, Minkah N, Kirn T, Troy A et al. (2009) Community-wide response of the gut microbiota to enteropathogenic *Citrobacter* *rodentium* infection revealed by deep sequencing. Infect Immun 77: 4668-4678. doi:10.1128/IAI.00493-09. PubMed: 19635824. 19635824 10.1128/IAI.00493-09 PMC2747949

[68] Symonds EL, Riedel CU, O’Mahony D, Lapthorne S, O’Mahony L et al. (2009) Involvement of T helper type 17 and regulatory T cell activity in Citrobacter rodentium invasion and inflammatory damage. Clin Exp Immunol 157: 148-154. doi:10.1111/j.1365-2249.2009.03934.x. PubMed: 19659780. 19659780 10.1111/j.1365-2249.2009.03934.x PMC2710602

[69] McBee ME, Zheng PZ, Rogers AB, Fox JG, Schauer DB (2008) Modulation of acute diarrheal illness by persistent bacterial infection. Infect Immun 76: 4851-4858. doi:10.1128/IAI.00745-08. PubMed: 18710857. 18710857 10.1128/IAI.00745-08 PMC2573363

[70] Karlsson J, Pütsep K, Chu H, Kays RJ, Bevins CL et al. (2008) Regional variations in Paneth cell antimicrobial peptide expression along the mouse intestinal tract. BMC Immunol 9: 37. doi:10.1186/1471-2172-9-37. PubMed: 18637162. 18637162 10.1186/1471-2172-9-37 PMC2488317

[71] Biswas A, Liu YJ, Hao L, Mizoguchi A, Salzman NH et al. (2010) Induction and rescue of Nod2-dependent Th1-driven granulomatous inflammation of the ileum. Proc Natl Acad Sci U S A 107: 14739-14744. doi:10.1073/pnas.1003363107. PubMed: 20679225. 20679225 10.1073/pnas.1003363107 PMC2930434

[72] Andersson ML, Karlsson-Sjöberg JM, Pütsep KL (2012) CRS-peptides: unique defense peptides of mouse Paneth cells. Mucosal Immunol 5: 367-376. doi:10.1038/mi.2012.22. PubMed: 22535181. 22535181 10.1038/mi.2012.22

[73] Mastroianni JR, Ouellette AJ (2009) Alpha-defensins in enteric innate immunity: functional Paneth cell alpha-defensins in mouse colonic lumen. J Biol Chem 284: 27848-27856. doi:10.1074/jbc.M109.050773. PubMed: 19687006. 19687006 10.1074/jbc.M109.050773 PMC2788835

[74] Haag LM, Fischer A, Otto B, Plickert R, Kühl AA et al. (2012) *Campylobacter* *jejuni* induces acute enterocolitis in gnotobiotic IL-10-/- mice via Toll-like-receptor-2 and -4 signaling. PLOS ONE 7: e40761. doi:10.1371/journal.pone.0040761. PubMed: 22808254. 22808254 10.1371/journal.pone.0040761 PMC3393706

[75] Siegesmund AM, Konkel ME, Klena JD, Mixter PF (2004) *Campylobacter* *jejuni* infection of differentiated THP-1 macrophages results in interleukin 1β release and caspase-1-independent apoptosis. Microbiology 150: 561-569. doi:10.1099/mic.0.26466-0. PubMed: 14993305. 14993305 10.1099/mic.0.26466-0

[76] Shi J, Aono S, Lu W, Ouellette AJ, Hu X et al. (2007) A novel role for defensins in intestinal homeostasis: regulation of IL-1β secretion. J Immunol 179: 1245-1253. PubMed: 17617617. 17617617 10.4049/jimmunol.179.2.1245

[77] Thiennimitr P, Winter SE, Winter MG, Xavier MN, Tolstikov V et al. (2011) Intestinal inflammation allows *Salmonella* to use ethanolamine to compete with the microbiota. Proc Natl Acad Sci U S A 108: 17480-17485. doi:10.1073/pnas.1107857108. PubMed: 21969563. 21969563 10.1073/pnas.1107857108 PMC3198331

[78] Winter SE, Thiennimitr P, Winter MG, Butler BP, Huseby DL et al. (2010) Gut inflammation provides a respiratory electron acceptor for *Salmonella* . Nature 467: 426-429. doi:10.1038/nature09415. PubMed: 20864996. 20864996 10.1038/nature09415 PMC2946174

[79] Stecher B, Robbiani R, Walker AW, Westendorf AM, Barthel M et al. (2007) *Salmonella* *enterica* serovar Typhimurium exploits inflammation to compete with the intestinal microbiota. PLOS Biol 5: 2177-2189. PubMed: 17760501. 17760501 10.1371/journal.pbio.0050244 PMC1951780

[80] Bavananthasivam J, Dassanayake RP, Kugadas A, Shanthalingam S, Call DR et al. (2012) Proximity-dependent inhibition of growth of *Mannheimia* *haemolytica* by *Pasteurella* *multocida* . Appl Environ Microbiol 78: 6683-6688. doi:10.1128/AEM.01119-12. PubMed: 22798357. 22798357 10.1128/AEM.01119-12 PMC3426682

[81] Songer JG, Post WK (2005) The genus Brachyspira Veterinary Microbiology: Bacterial and Fungal Agents of Animal Diseases. 1st ed. St. Louis, MO: Elsevier Saunders pp. 232-239.

[82] Quinn PJ, Markey BK, Leonard FC, FitzPatrik ES, Fanning S et al. (2011) *Lawsonia**intracellularis*. Veterinary Microbiology and Microbial Diseases. 2nd ed. West Sussex: Wiley-Blackwell, UK. pp. 351-353

[83] Quinn PJ, Markey BK, Leonard FC, FitzPatrik ES, Fanning S et al. (2011) Pathogenic anaerobic non-spore-forming Gram-negative bacteria. Veterinary Microbiology and Microbial Diseases. 2nd ed. West Sussex, UK: Wiley-Blackwell pp. 367-371.

