# Supplementary material for: Campylobacter jejuni Colonization Is Associated with a Dysbiosis in the Cecal Microbiota of Mice in the Absence of Prominent Inflammation
Source: PLoS One. 2013 Sep 16;8(9):e75325. doi: 10.1371/journal.pone.0075325 (PMC3774657; doi:10.1371/journal.pone.0075325)
Supplement: Table S1 — Identities of operational taxonomic units (OTUs). See Figure 6 for the relative frequency of individual OTUs by sample within treatments (i.e. OTUs for which ten sequences or more were observed). (DOCX) [file pone.0075325.s001.docx]

**Table S1.** Identities of operational taxonomic units (OTUs).

| **OTU #** | **Identity** |
| --- | --- |
| 0 | Bacteria; Firmicutes; Clostridia; Clostridiales; Lachnospiraceae |
| 1 | Bacteria; Firmicutes; Clostridia; Clostridiales; Lachnospiraceae |
| 2 | Bacteria; Firmicutes; Clostridia; Clostridiales; Lachnospiraceae |
| 3 | Bacteria; Firmicutes; Clostridia; Clostridiales; Ruminococcaceae; *Oscillospira* |
| 4 | Bacteria; Firmicutes; Clostridia; Clostridiales; Lachnospiraceae |
| 5 | Bacteria; Firmicutes; Clostridia; Clostridiales; Lachnospiraceae |
| 6 | Bacteria; Firmicutes; Clostridia; Clostridiales; Ruminococcaceae |
| 7 | Bacteria; Firmicutes; Clostridia; Clostridiales; Lachnospiraceae |
| 8 | Bacteria; Firmicutes; Clostridia; Clostridiales; Ruminococcaceae; *Ruminococcus* |
| 9 | Bacteria; Firmicutes; Clostridia |
| 10 | Bacteria; Firmicutes; Clostridia; Clostridiales; Lachnospiraceae |
| 11 | Bacteria; Firmicutes; Clostridia; Clostridiales |
| 12 | Bacteria; Firmicutes; Clostridia; Clostridiales; Lachnospiraceae |
| 13 | Bacteria; Firmicutes; Clostridia; Clostridiales; Lachnospiraceae |
| 14 | Bacteria; Firmicutes; Clostridia; Clostridiales; Lachnospiraceae |
| 15 | Bacteria; Firmicutes; Clostridia; Clostridiales; Ruminococcaceae |
| 16 | Bacteria; Bacteroidetes; Bacteroidia; Bacteroidales |
| 17 | Bacteria; Tenericutes; Mollicutes |
| 18 | Bacteria; Firmicutes; Clostridia; Clostridiales; Lachnospiraceae |
| 19 | Bacteria |
| 20 | Bacteria; Firmicutes; Clostridia; Clostridiales; Ruminococcaceae; *Oscillospira* |
| 21 | Bacteria; Firmicutes; Clostridia; Clostridiales; Lachnospiraceae |
| 22 | Bacteria; Firmicutes; Clostridia; Clostridiales; Lachnospiraceae |
| 23 | Bacteria; Firmicutes; Clostridia; Clostridiales; Ruminococcaceae; *Oscillospira* |
| 24 | Bacteria; Bacteroidetes; Bacteroidia; Bacteroidales |
| 25 | Bacteria; Firmicutes; Clostridia; Clostridiales; Lachnospiraceae |
| 26 | Bacteria; Firmicutes; Clostridia; Clostridiales; Lachnospiraceae |
| 27 | Bacteria; Firmicutes; Clostridia |
| 28 | Bacteria; Proteobacteria; Deltaproteobacteria |
| 29 | Bacteria; Firmicutes; Clostridia; Clostridiales; Lachnospiraceae |
| 30 | Bacteria; Firmicutes; Clostridia; Clostridiales; Lachnospiraceae |
| 31 | Bacteria; Firmicutes; Clostridia; Clostridiales; Ruminococcaceae |
| 32 | Bacteria; Firmicutes; Clostridia; Clostridiales; Lachnospiraceae |
| 33 | Bacteria; Firmicutes; Clostridia; Clostridiales; Ruminococcaceae; *Oscillospira* |
| 34 | Bacteria; Firmicutes; Clostridia; Clostridiales; Lachnospiraceae |
| 35 | Bacteria; Firmicutes; Clostridia; Clostridiales; Lachnospiraceae |
| 36 | Bacteria; Firmicutes; Clostridia; Clostridiales; Ruminococcaceae |
| 37 | Bacteria; Firmicutes |
| 38 | Bacteria; Firmicutes; Clostridia; Clostridiales; Ruminococcaceae; *Oscillospira* |
| 39 | Bacteria; Firmicutes; Clostridia; Clostridiales; Lachnospiraceae |
| 40 | Bacteria; Firmicutes |
| 41 | Bacteria; Firmicutes; Clostridia; Clostridiales; Ruminococcaceae; *Ruminococcus* |
| 42 | Bacteria; Firmicutes; Clostridia; Clostridiales; Ruminococcaceae; *Oscillospira* |
| 43 | Bacteria; Firmicutes; Clostridia; Clostridiales; Lachnospiraceae |
| 44 | Bacteria; Firmicutes; Clostridia; Coriobacteriales; Coriobacteriaceae; *Adlercreutzia* |
| 45 | Bacteria; Firmicutes; Clostridia; Clostridiales; Lachnospiraceae |
| 46 | Bacteria; Firmicutes; Clostridia; Clostridiales; Lachnospiraceae |
| 47 | Bacteria; Firmicutes; Clostridia; Clostridiales; Lachnospiraceae |
| 48 | Bacteria; Firmicutes; Clostridia; Clostridiales; Lachnospiraceae; [*Ruminococcus*] *gnavus* |
| 49 | Bacteria; Firmicutes; Erysipelotrichi; Erysipelotrichales; Erysipelotrichaceae |
| 50 | Bacteria; Firmicutes; Clostridia; Clostridiales; Lachnospiraceae |
| 51 | Bacteria; Firmicutes; Clostridia; Clostridiales; Ruminococcaceae |
| 52 | Bacteria; Firmicutes; Clostridia; Clostridiales; Lachnospiraceae |
| 53 | Bacteria; Firmicutes; Clostridia; Clostridiales; Lachnospiraceae |
| 54 | Bacteria; Firmicutes; Clostridia |
| 55 | Bacteria; Firmicutes; Clostridia; Clostridiales; Ruminococcaceae |
| 56 | Bacteria; Firmicutes; Clostridia; Clostridiales; Lachnospiraceae |
| 57 | Bacteria; Firmicutes; Clostridia; Clostridiales; Lachnospiraceae |
| 58 | Bacteria; Firmicutes; Clostridia; Clostridiales; Lachnospiraceae |
| 59 | Bacteria; Firmicutes; Clostridia |
| 60 | Bacteria; Firmicutes; Clostridia; Clostridiales; Ruminococcaceae; *Oscillospira* |
| 61 | Bacteria; Firmicutes; Clostridia; Clostridiales; Lachnospiraceae |
| 62 | Bacteria; Firmicutes; Clostridia; Clostridiales; Lachnospiraceae |
| 63 | Bacteria; Firmicutes; Clostridia; Clostridiales; Ruminococcaceae |
| 64 | Bacteria; Firmicutes; Clostridia |
| 65 | Bacteria; Firmicutes; Clostridia; Clostridiales; Ruminococcaceae |
| 66 | Bacteria; Firmicutes; Clostridia; Clostridiales; Lachnospiraceae |
| 67 | Bacteria; Firmicutes; Clostridia; Clostridiales; Lachnospiraceae |
| 68 | Bacteria; Firmicutes; Clostridia; Clostridiales; Lachnospiraceae |
| 69 | Bacteria; Firmicutes; Clostridia; Clostridiales; Lachnospiraceae |
| 70 | Bacteria; Firmicutes; Clostridia; Clostridiales; Lachnospiraceae |
| 71 | Bacteria; Firmicutes; Clostridia; Clostridiales; Lachnospiraceae |
| 72 | Bacteria; Firmicutes; Clostridia; Clostridiales; Ruminococcaceae; *Oscillospira* |
| 73 | Bacteria; Firmicutes; Bacilli; Bacillales; Planococcaceae; *Lysinibacillus boronitolerans* |
| 74 | Bacteria; Firmicutes; Clostridia |
| 75 | Bacteria |
| 76 | Bacteria; Firmicutes; Clostridia; Clostridiales; Lachnospiraceae; [*Ruminococcus*] *gnavus* |
| 77 | Bacteria; Tenericutes; Mollicutes; Anaeroplasmatales; Anaeroplasmataceae; *Anaeroplasma* |
| 78 | Bacteria; Firmicutes; Clostridia; Clostridiales; Ruminococcaceae |
| 79 | Bacteria; Firmicutes; Clostridia; Clostridiales; Lachnospiraceae |
| 80 | Bacteria; Firmicutes; Clostridia; Clostridiales |
| 81 | Bacteria; Firmicutes; Clostridia; Clostridiales; Lachnospiraceae |
| 82 | Bacteria; Bacteroidetes; Bacteroidia; Bacteroidales |
| 83 | Bacteria; Firmicutes; Clostridia; Clostridiales; Lachnospiraceae |
| 84 | Bacteria; Firmicutes; Clostridia; Clostridiales; Lachnospiraceae |
| 85 | Bacteria; Firmicutes; Clostridia; Clostridiales; Lachnospiraceae |
| 86 | Bacteria; Firmicutes; Clostridia; Clostridiales; Lachnospiraceae |
| 87 | Bacteria; Firmicutes; Clostridia; Clostridiales; Lachnospiraceae; [*Ruminococcus*] *gnavus* |
| 88 | Bacteria; Firmicutes; Clostridia |
| 89 | Bacteria |
| 90 | Bacteria; Firmicutes; Clostridia; Clostridiales; Lachnospiraceae |
| 91 | Bacteria; Firmicutes; Clostridia; Clostridiales; Ruminococcaceae; *Ruminococcus* |
| 92 | Bacteria; Firmicutes; Clostridia; Clostridiales; Lachnospiraceae |
| 93 | Bacteria; Firmicutes |
| 94 | Bacteria; Firmicutes; Clostridia; Clostridiales; Lachnospiraceae; [*Ruminococcus*] *gnavus* |
| 95 | Bacteria; Firmicutes; Erysipelotrichi; Erysipelotrichales; Erysipelotrichaceae |
| 96 | Bacteria; Firmicutes; Clostridia; Clostridiales; Lachnospiraceae |
| 97 | Bacteria; Firmicutes; Clostridia; Clostridiales; Lachnospiraceae |
| 98 | Bacteria; Firmicutes; Bacilli; Turicibacterales; Turicibacteraceae; *Turicibacter* |
| 99 | Bacteria; Firmicutes |
| 100 | Bacteria; Firmicutes; Clostridia; Clostridiales; Lachnospiraceae |
| 101 | Bacteria; Firmicutes; Clostridia; Clostridiales; Lachnospiraceae |
| 102 | Bacteria; Firmicutes; Clostridia; Clostridiales; Ruminococcaceae |
| 103 | Bacteria; Firmicutes; Clostridia; Clostridiales |
| 104 | Bacteria; Firmicutes; Clostridia; Clostridiales; Lachnospiraceae |
| 105 | Bacteria; Firmicutes; Clostridia; Clostridiales; Lachnospiraceae |
| 106 | Bacteria; Firmicutes; Clostridia; Clostridiales; Lachnospiraceae |
| 107 | Bacteria; Firmicutes; Clostridia; Clostridiales; Ruminococcaceae |
| 108 | Bacteria |
| 109 | Bacteria |
| 110 | Bacteria; Firmicutes; Clostridia; Clostridiales; Lachnospiraceae |
| 111 | Bacteria; Firmicutes; Clostridia; Clostridiales; Ruminococcaceae |
| 112 | Bacteria; Firmicutes; Clostridia; Clostridiales; Lachnospiraceae |
| 113 | Bacteria; Firmicutes; Clostridia; Clostridiales; Ruminococcaceae; *Oscillospira* |
| 114 | Bacteria; Firmicutes; Clostridia; Clostridiales; Ruminococcaceae |
| 115 | Bacteria; Firmicutes; Clostridia; Clostridiales; Ruminococcaceae |
| 116 | Bacteria; Firmicutes; Clostridia; Clostridiales; Lachnospiraceae |
| 117 | Bacteria; Firmicutes; Clostridia; Clostridiales; Lachnospiraceae |
| 118 | Bacteria; Firmicutes; Clostridia; Clostridiales; Lachnospiraceae |
| 119 | Bacteria; Firmicutes; Clostridia; Clostridiales; Lachnospiraceae |
| 120 | Bacteria; Firmicutes; Clostridia; Clostridiales |
| 121 | Bacteria; Firmicutes; Clostridia; Clostridiales; Ruminococcaceae |
| 122 | Bacteria; Firmicutes; Clostridia; Clostridiales; Lachnospiraceae |
| 123 | Bacteria; Firmicutes; Clostridia; Clostridiales; Ruminococcaceae; *Oscillospira* |
| 124 | Bacteria; Firmicutes; Clostridia; Clostridiales; Ruminococcaceae; *Oscillospira* |
| 125 | Bacteria; Firmicutes; Clostridia; Clostridiales; Lachnospiraceae |
| 126 | Bacteria; Firmicutes; Clostridia; Clostridiales; Clostridiaceae |
| 127 | Bacteria; Firmicutes; Clostridia; Clostridiales; Ruminococcaceae |
| 128 | Bacteria; Firmicutes; Clostridia; Clostridiales; Lachnospiraceae |
| 129 | Bacteria; Firmicutes; Clostridia; Clostridiales; Lachnospiraceae |
| 130 | Bacteria; Firmicutes; Clostridia |
| 131 | Bacteria; Firmicutes; Clostridia; Clostridiales; Lachnospiraceae |
| 132 | Bacteria; Firmicutes; Clostridia; Clostridiales; Lachnospiraceae |
| 133 | Bacteria; Firmicutes; Clostridia; Coriobacteriales; Coriobacteriaceae; *Adlercreutzia* |
| 134 | Bacteria; Firmicutes; Clostridia; Clostridiales; Ruminococcaceae |
| 135 | Bacteria; Firmicutes; Clostridia; Clostridiales; Lachnospiraceae |
| 136 | Bacteria; Firmicutes; Clostridia; Clostridiales |
| 137 | Bacteria; Firmicutes; Clostridia; Clostridiales; Ruminococcaceae |
| 138 | Bacteria; Proteobacteria; Betaproteobacteria; Burkholderiales; Oxalobacteraceae; *Herbaspirillum* |
| 139 | Bacteria; Firmicutes; Clostridia; Clostridiales; Lachnospiraceae |
| 140 | Bacteria; Firmicutes; Clostridia; Clostridiales; Lachnospiraceae |
| 141 | Bacteria; Firmicutes; Clostridia; Clostridiales; Clostridiaceae |
| 142 | Bacteria; Firmicutes; Clostridia; Clostridiales; Lachnospiraceae |
| 143 | Bacteria; Firmicutes; Erysipelotrichi; Erysipelotrichales; [Coprobacillaceae] |
| 144 | Bacteria; Firmicutes |
| 145 | Bacteria; Bacteroidetes; Bacteroidia; Bacteroidales |
| 146 | Bacteria; Firmicutes; Clostridia; Clostridiales |
| 147 | Bacteria; Firmicutes; Clostridia; Clostridiales; Lachnospiraceae |
| 148 | Bacteria; Firmicutes; Clostridia; Clostridiales; Lachnospiraceae |
| 149 | Bacteria; Firmicutes; Clostridia; Clostridiales |
| 150 | Bacteria; Firmicutes; Clostridia; Clostridiales; Ruminococcaceae; *Oscillospira* |
| 151 | Bacteria; Proteobacteria; Alphaproteobacteria; Rhodobacterales; Rhodobacteraceae |
| 152 | Bacteria; Firmicutes |
| 153 | Bacteria; Firmicutes; Clostridia; Clostridiales; Lachnospiraceae |
| 154 | Bacteria; Firmicutes; Clostridia; Clostridiales |
| 155 | Bacteria |
| 156 | Bacteria; Firmicutes; Clostridia; Clostridiales; Lachnospiraceae |
| 157 | Bacteria; Firmicutes; Clostridia; Clostridiales; Ruminococcaceae; *Ruminococcus* |
| 158 | Bacteria; Firmicutes; Clostridia; Clostridiales; Lachnospiraceae |
| 159 | Bacteria; Firmicutes; Clostridia; Clostridiales; Lachnospiraceae |
| 160 | Bacteria; Firmicutes; Clostridia; Clostridiales; Lachnospiraceae |
| 161 | Bacteria; Firmicutes; Clostridia; Clostridiales; Lachnospiraceae |
| 162 | Bacteria; Firmicutes; Clostridia; Clostridiales; Ruminococcaceae |
| 163 | Bacteria; Firmicutes; Clostridia; Clostridiales; Lachnospiraceae |
| 164 | Bacteria; Firmicutes; Clostridia; Clostridiales; Lachnospiraceae |
| 165 | Bacteria; Firmicutes; Clostridia; Clostridiales; Lachnospiraceae; [*Ruminococcus*] *gnavus* |
| 166 | Bacteria; Firmicutes; Clostridia; Clostridiales; Lachnospiraceae |
| 167 | Bacteria; Firmicutes; Clostridia; Clostridiales; Lachnospiraceae; *Anaerostipes* |
| 168 | Bacteria; Firmicutes; Clostridia; Clostridiales; Lachnospiraceae |
| 169 | Bacteria; Firmicutes; Clostridia; Clostridiales; Lachnospiraceae |
| 170 | Bacteria; Firmicutes; Clostridia; Clostridiales; Dehalobacteriaceae; *Dehalobacterium* |
| 171 | Bacteria; Firmicutes; Clostridia; Clostridiales; Lachnospiraceae; [*Ruminococcus*] *gnavus* |
| 172 | Bacteria; Firmicutes; Clostridia; Clostridiales; Lachnospiraceae |
| 173 | Bacteria; Firmicutes; Clostridia; Clostridiales; Lachnospiraceae |
| 174 | Bacteria; Firmicutes; Clostridia; Clostridiales |
| 175 | Bacteria; Firmicutes; Clostridia; Clostridiales; Lachnospiraceae |
| 176 | Bacteria; Firmicutes; Clostridia; Clostridiales; Ruminococcaceae; *Oscillospira* |
| 177 | Bacteria; Firmicutes; Clostridia |
| 178 | Bacteria; Verrucomicrobia; Opitutae; Puniceicoccales; Puniceicoccaceae |
| 179 | Bacteria; Firmicutes; Clostridia; Clostridiales; Lachnospiraceae |
| 180 | Bacteria; Firmicutes; Clostridia; Clostridiales; Lachnospiraceae; [*Ruminococcus*] *gnavus* |
| 181 | Bacteria; Firmicutes; Clostridia; Clostridiales; Lachnospiraceae |
| 182 | Bacteria; Firmicutes; Clostridia; Clostridiales; Lachnospiraceae |
| 183 | Bacteria; Firmicutes; Clostridia; Clostridiales; Lachnospiraceae |
| 184 | Bacteria; Firmicutes; Clostridia; Clostridiales; Lachnospiraceae |
| 185 | Bacteria; Firmicutes; Clostridia; Clostridiales |
| 186 | Bacteria; Firmicutes; Clostridia; Clostridiales; Lachnospiraceae |
| 187 | Bacteria; Firmicutes; Clostridia; Clostridiales; Lachnospiraceae; [*Ruminococcus*] *gnavus* |
| 188 | Bacteria; Firmicutes; Clostridia; Clostridiales |
| 189 | Bacteria; Firmicutes; Clostridia; Clostridiales; Lachnospiraceae |
| 190 | Bacteria |
| 191 | Bacteria; Firmicutes; Clostridia; Clostridiales; Lachnospiraceae |
| 192 | Bacteria; Firmicutes; Bacilli; Lactobacillales; Lactobacillaceae; *Lactobacillus* |
| 193 | Bacteria; Firmicutes; Clostridia; Clostridiales; Lachnospiraceae |
| 194 | Bacteria; Firmicutes; Clostridia; Clostridiales; Lachnospiraceae |
| 195 | Bacteria; Firmicutes; Clostridia; Clostridiales; Lachnospiraceae |
| 196 | Bacteria; Firmicutes; Clostridia; Clostridiales; Lachnospiraceae; [*Ruminococcus*] *gnavus* |
| 197 | Bacteria; Bacteroidetes; Bacteroidia; Bacteroidales |
| 198 | Bacteria; Firmicutes; Clostridia; Clostridiales; Lachnospiraceae |
| 199 | Bacteria; Firmicutes; Clostridia; Clostridiales; Lachnospiraceae |
| 200 | Bacteria; Firmicutes |
| 201 | Bacteria; Firmicutes; Clostridia; Clostridiales; Lachnospiraceae; [*Ruminococcus*] *gnavus* |
| 202 | Bacteria; Firmicutes; Clostridia; Clostridiales; Lachnospiraceae |
| 203 | Bacteria; Firmicutes; Clostridia; Clostridiales; Lachnospiraceae |
| 204 | Bacteria; Firmicutes; Clostridia; Clostridiales; Ruminococcaceae |
| 205 | Bacteria; Firmicutes; Clostridia; Clostridiales; Lachnospiraceae |
| 206 | Bacteria |
| 207 | Bacteria; Firmicutes; Clostridia; Clostridiales; Ruminococcaceae; *Oscillospira* |
| 208 | Bacteria; Firmicutes; Clostridia; Clostridiales; Ruminococcaceae; *Oscillospira* |
| 209 | Bacteria; Firmicutes; Clostridia; Clostridiales; Lachnospiraceae |
| 210 | Bacteria; Firmicutes; Clostridia; Clostridiales; Ruminococcaceae |
| 211 | Bacteria; Firmicutes; Clostridia; Clostridiales; Lachnospiraceae |
| 212 | Bacteria; Proteobacteria; Gammaproteobacteria; Xanthomonadales; Xanthomonadaceae; *Stenotrophomonas geniculata* |
| 213 | Bacteria; Bacteroidetes; Bacteroidia; Bacteroidales |
| 214 | Bacteria; Firmicutes; Clostridia; Clostridiales; Lachnospiraceae |
| 215 | Bacteria; Firmicutes; Clostridia; Clostridiales; Lachnospiraceae |
| 216 | Bacteria; Firmicutes; Clostridia; Clostridiales; Lachnospiraceae |
| 217 | Bacteria; Firmicutes; Clostridia; Clostridiales; Lachnospiraceae |
| 218 | Bacteria; Firmicutes; Clostridia; Clostridiales; Ruminococcaceae |
| 219 | Bacteria; Firmicutes; Clostridia; Clostridiales; Lachnospiraceae |
| 220 | Bacteria; Firmicutes; Clostridia; Clostridiales; Ruminococcaceae |
| 221 | Bacteria; Firmicutes; Clostridia; Clostridiales; Lachnospiraceae; [*Ruminococcus*] *gnavus* |
| 222 | Bacteria; Firmicutes; Clostridia; Clostridiales; Lachnospiraceae |
| 223 | Bacteria; Firmicutes; Clostridia; Clostridiales; Lachnospiraceae |
| 224 | Bacteria; Firmicutes; Clostridia; Clostridiales; Lachnospiraceae |
| 225 | Bacteria; Firmicutes; Clostridia; Clostridiales; Lachnospiraceae |
| 226 | Bacteria; Firmicutes; Clostridia; Clostridiales; Lachnospiraceae |
| 227 | Bacteria; Firmicutes; Clostridia; Clostridiales; Lachnospiraceae |
| 228 | Bacteria; Firmicutes; Clostridia; Clostridiales; Lachnospiraceae |
| 229 | Bacteria; Firmicutes; Clostridia; Clostridiales; Lachnospiraceae |
| 230 | Bacteria; Firmicutes; Clostridia; Clostridiales; Ruminococcaceae; *Oscillospira* |
| 231 | Bacteria; Bacteroidetes; Bacteroidia; Bacteroidales |
| 232 | Bacteria; Firmicutes; Clostridia; Clostridiales; Lachnospiraceae |
| 233 | Bacteria; Firmicutes; Clostridia; Clostridiales; Lachnospiraceae; [*Ruminococcus*] *gnavus* |
| 234 | Bacteria; Firmicutes; Clostridia; Clostridiales; Lachnospiraceae |
| 235 | Bacteria; Firmicutes; Clostridia; Clostridiales; Lachnospiraceae |
| 236 | Bacteria; Firmicutes; Clostridia |
| 237 | Bacteria; Firmicutes; Clostridia; Clostridiales; Lachnospiraceae |
| 238 | Bacteria; Firmicutes; Clostridia; Clostridiales; Ruminococcaceae; *Oscillospira* |
| 239 | Bacteria; Firmicutes; Clostridia; Clostridiales; Ruminococcaceae; *Ruminococcus* |
| 240 | Bacteria; Firmicutes; Clostridia; Clostridiales; Lachnospiraceae |
| 241 | Bacteria; Firmicutes; Clostridia; Clostridiales; Lachnospiraceae |
| 242 | Bacteria; Firmicutes; Clostridia; Clostridiales; Ruminococcaceae |
| 243 | Bacteria; Firmicutes; Clostridia; Clostridiales; Lachnospiraceae |
| 244 | Bacteria; Firmicutes; Clostridia; Clostridiales; Lachnospiraceae |
| 245 | Bacteria; Bacteroidetes; Flavobacteriia; Flavobacteriales; Flavobacteriaceae |
| 246 | Bacteria; Firmicutes; Clostridia; Clostridiales |
| 247 | Bacteria; Firmicutes |
| 248 | Bacteria; Firmicutes; Clostridia; Clostridiales; Ruminococcaceae |
| 249 | Bacteria; Firmicutes; Clostridia; Clostridiales; Lachnospiraceae |
| 250 | Bacteria; Firmicutes; Clostridia |
| 251 | Bacteria; Firmicutes; Clostridia; Clostridiales; Lachnospiraceae |
| 252 | Bacteria |
| 253 | Bacteria; Firmicutes; Clostridia; Clostridiales; Lachnospiraceae |
| 254 | Bacteria; Firmicutes; Clostridia; Clostridiales; Lachnospiraceae |
| 255 | Bacteria; Firmicutes; Clostridia; Clostridiales; Ruminococcaceae; *Oscillospira* |
| 256 | Bacteria; Firmicutes; Clostridia; Clostridiales; Lachnospiraceae |
| 257 | Bacteria; Firmicutes; Clostridia; Clostridiales; Ruminococcaceae |
| 258 | Bacteria; Firmicutes; Clostridia; Clostridiales; Lachnospiraceae |
| 259 | Bacteria; Firmicutes; Clostridia |
| 260 | Bacteria; Firmicutes; Clostridia; Clostridiales; Ruminococcaceae; *Ruminococcus* |
| 261 | Bacteria; Firmicutes; Clostridia; Clostridiales; Lachnospiraceae; [*Ruminococcus*] *gnavus* |
| 262 | Bacteria; Firmicutes; Clostridia; Clostridiales; Lachnospiraceae |
| 263 | Bacteria; Firmicutes; Clostridia; Clostridiales; Lachnospiraceae |
| 264 | Bacteria; Firmicutes; Clostridia |
| 265 | Bacteria; Firmicutes; Clostridia; Clostridiales; Ruminococcaceae; *Anaerotruncus* |
| 266 | Bacteria; Firmicutes; Clostridia; Clostridiales; Lachnospiraceae; [*Ruminococcus*] *gnavus* |
| 267 | Bacteria; Bacteroidetes; Bacteroidia; Bacteroidales |
| 268 | Bacteria |
| 269 | Bacteria; Firmicutes; Clostridia; Clostridiales; Ruminococcaceae; *Oscillospira* |
| 270 | Bacteria; Firmicutes; Clostridia; Clostridiales |
| 271 | Bacteria; Bacteroidetes; Bacteroidia; Bacteroidales |
| 272 | Bacteria; Firmicutes; Clostridia; Clostridiales; Lachnospiraceae |
| 273 | Bacteria; Firmicutes; Clostridia; Clostridiales; Lachnospiraceae |
| 274 | Bacteria; Firmicutes; Clostridia |
| 275 | Bacteria; Firmicutes; Clostridia; Clostridiales; Ruminococcaceae |
| 276 | Bacteria; Firmicutes; Clostridia; Clostridiales; Lachnospiraceae |
| 277 | Bacteria; Firmicutes; Clostridia; Clostridiales; Lachnospiraceae |
| 278 | Bacteria; Firmicutes; Bacilli; Lactobacillales; Lactobacillaceae |
| 279 | Bacteria; Firmicutes; Clostridia; Clostridiales; Lachnospiraceae; [*Ruminococcus*] *gnavus* |
| 280 | Bacteria; Firmicutes; Clostridia; Clostridiales |
| 281 | Bacteria; Firmicutes; Clostridia; Clostridiales; Lachnospiraceae |
| 282 | Bacteria; Firmicutes; Clostridia; Clostridiales; Lachnospiraceae |
| 283 | Bacteria |
| 284 | Bacteria; Actinobacteria; Actinobacteria; Actinomycetales; Propionibacteriaceae; *Propionibacterium granulosum* |
| 285 | Bacteria; Firmicutes; Clostridia |
| 286 | Bacteria; Firmicutes; Clostridia; Clostridiales; Ruminococcaceae; *Oscillospira* |
| 287 | Bacteria; Firmicutes |
| 288 | Bacteria; Firmicutes; Clostridia; Clostridiales |
| 289 | Bacteria; Firmicutes; Clostridia; Clostridiales; Ruminococcaceae; *Oscillospira* |
| 290 | Bacteria; Firmicutes; Clostridia; Clostridiales; Lachnospiraceae |
| 291 | Bacteria; Firmicutes; Clostridia; Clostridiales; Ruminococcaceae |
| 292 | Bacteria; Firmicutes; Clostridia; Clostridiales; Lachnospiraceae |
| 293 | Bacteria; Firmicutes; Clostridia; Clostridiales; Lachnospiraceae |
| 294 | Bacteria; Firmicutes; Clostridia; Clostridiales; Lachnospiraceae |
| 295 | Bacteria; Firmicutes; Clostridia; Clostridiales; Ruminococcaceae; *Oscillospira* |
| 296 | Bacteria; Firmicutes; Clostridia; Clostridiales |
| 297 | Bacteria; Firmicutes; Clostridia |
| 298 | Bacteria; Firmicutes; Clostridia; Clostridiales; Lachnospiraceae |
| 299 | Bacteria; Firmicutes; Clostridia; Clostridiales; Lachnospiraceae |
| 300 | Bacteria; Firmicutes; Clostridia; Clostridiales; Ruminococcaceae; *Oscillospira* |
| 301 | Bacteria; Firmicutes; Clostridia; Clostridiales; Lachnospiraceae |
| 302 | Bacteria; Firmicutes; Clostridia; Clostridiales; Ruminococcaceae; *Oscillospira* |
| 303 | Bacteria; Firmicutes; Clostridia; Clostridiales; Lachnospiraceae |
| 304 | Bacteria; Firmicutes; Clostridia; Clostridiales; Lachnospiraceae |
| 305 | Bacteria; Firmicutes; Clostridia; Clostridiales; Lachnospiraceae |
| 306 | Bacteria; Firmicutes; Clostridia; Clostridiales; Lachnospiraceae |
| 307 | Bacteria; Firmicutes; Clostridia; Clostridiales; Lachnospiraceae |
| 308 | Bacteria |
| 309 | Bacteria; Firmicutes; Clostridia; Clostridiales; Lachnospiraceae |
| 310 | Bacteria; Firmicutes; Clostridia; Clostridiales; Ruminococcaceae |
| 311 | Bacteria; Firmicutes; Clostridia; Clostridiales; Lachnospiraceae |
| 312 | Bacteria; Firmicutes; Clostridia; Clostridiales; Lachnospiraceae; [*Ruminococcus*] *gnavus* |
| 313 | Bacteria; Firmicutes; Clostridia; Clostridiales; Lachnospiraceae; [*Ruminococcus*] *gnavus* |
| 314 | Bacteria; Firmicutes; Erysipelotrichi; Erysipelotrichales; Erysipelotrichaceae |
| 315 | Bacteria; Firmicutes; Clostridia; Clostridiales |
| 316 | Bacteria; Proteobacteria; Alphaproteobacteria; Rickettsiales; Pelagibacteraceae |
| 317 | Bacteria; Firmicutes; Clostridia; Clostridiales; Lachnospiraceae |
| 318 | Bacteria; Firmicutes; Clostridia; Clostridiales; Lachnospiraceae |
| 319 | Bacteria; Firmicutes; Clostridia; Clostridiales |
| 320 | Bacteria; Proteobacteria; Epsilonproteobacteria; Campylobacterales; Campylobacteraceae; *Campylobacter* |
| 321 | Bacteria; Firmicutes; Clostridia; Clostridiales; Lachnospiraceae |
| 322 | Bacteria; Firmicutes; Bacilli; Lactobacillales; Lactobacillaceae |
| 323 | Bacteria; Firmicutes; Clostridia; Clostridiales; Lachnospiraceae; [*Ruminococcus*] *gnavus* |
| 324 | Bacteria; Firmicutes; Clostridia; Clostridiales; Lachnospiraceae |
| 325 | Bacteria; Firmicutes; Clostridia; Clostridiales; Lachnospiraceae |
| 326 | Bacteria; Firmicutes; Clostridia; Clostridiales; Lachnospiraceae |
| 327 | Bacteria; Firmicutes; Clostridia; Clostridiales; Lachnospiraceae |
| 328 | Bacteria; Firmicutes; Clostridia; Clostridiales; Ruminococcaceae; *Oscillospira* |
| 329 | Bacteria; Firmicutes; Clostridia; Clostridiales; Ruminococcaceae; *Oscillospira* |
| 330 | Bacteria; Firmicutes; Clostridia; Clostridiales; Lachnospiraceae |
| 331 | Bacteria; Firmicutes; Clostridia |
| 332 | Bacteria; Firmicutes; Clostridia; Clostridiales; Lachnospiraceae |
| 333 | Bacteria; Firmicutes; Clostridia; Clostridiales; Lachnospiraceae |
| 334 | Bacteria |
| 335 | Bacteria; Firmicutes; Clostridia; Clostridiales; Ruminococcaceae; *Oscillospira* |
| 336 | Bacteria; Firmicutes; Clostridia; Clostridiales; Ruminococcaceae; *Oscillospira* |
| 337 | Bacteria; Firmicutes; Clostridia; Clostridiales; Lachnospiraceae |
| 338 | Bacteria; Bacteroidetes; Bacteroidia; Bacteroidales |
| 339 | Bacteria; Firmicutes; Clostridia |
| 340 | Bacteria; Firmicutes; Clostridia; Clostridiales; Lachnospiraceae; [*Ruminococcus*] *gnavus* |
| 341 | Bacteria; Firmicutes; Clostridia; Clostridiales; Lachnospiraceae |
| 342 | Bacteria; Firmicutes; Clostridia; Coriobacteriales; Coriobacteriaceae; *Adlercreutzia* |
| 343 | Bacteria; Firmicutes; Clostridia; Clostridiales; Lachnospiraceae |
| 344 | Bacteria; Firmicutes; Clostridia; Clostridiales; Ruminococcaceae; *Oscillospira* |
| 345 | Bacteria; Firmicutes; Clostridia; Clostridiales; Lachnospiraceae |
| 346 | Bacteria; Firmicutes; Clostridia; Clostridiales; Lachnospiraceae |
| 347 | Bacteria; Firmicutes; Clostridia; Clostridiales; Ruminococcaceae |
| 348 | Bacteria; Tenericutes; Mollicutes |
| 349 | Bacteria |
| 350 | Bacteria; Firmicutes; Clostridia; Clostridiales; Lachnospiraceae |
| 351 | Bacteria; Firmicutes; Clostridia; Clostridiales; Lachnospiraceae |
| 352 | Bacteria; Firmicutes; Clostridia; Clostridiales; Lachnospiraceae |
| 353 | Bacteria; Firmicutes; Clostridia; Clostridiales; Lachnospiraceae |
| 354 | Bacteria; Firmicutes; Clostridia; Clostridiales; Ruminococcaceae |
| 355 | Bacteria; Firmicutes; Clostridia; Clostridiales; Lachnospiraceae |
| 356 | Bacteria; Firmicutes; Clostridia; Clostridiales; Ruminococcaceae |
| 357 | Bacteria; Bacteroidetes; Bacteroidia; Bacteroidales |
| 358 | Bacteria; Firmicutes; Clostridia; Clostridiales; Lachnospiraceae |
| 359 | Bacteria; Firmicutes; Clostridia; Clostridiales; Lachnospiraceae |
| 360 | Bacteria; Firmicutes; Clostridia; Clostridiales; Lachnospiraceae |
| 361 | Bacteria; Firmicutes; Clostridia; Clostridiales; Ruminococcaceae |
| 362 | Bacteria; Firmicutes; Clostridia |
| 363 | Bacteria; Firmicutes; Clostridia; Clostridiales; Lachnospiraceae |
| 364 | Bacteria; Firmicutes; Clostridia; Clostridiales; Lachnospiraceae |
| 365 | Bacteria; Firmicutes; Clostridia; Clostridiales; Lachnospiraceae |
| 366 | Bacteria; Firmicutes; Clostridia; Clostridiales; Ruminococcaceae; *Anaerotruncus* |
| 367 | Bacteria; Firmicutes; Clostridia; Clostridiales |
| 368 | Bacteria; Firmicutes; Clostridia |
| 369 | Bacteria; Firmicutes; Clostridia; Clostridiales; Lachnospiraceae |
| 370 | Bacteria; Firmicutes; Clostridia; Clostridiales; Lachnospiraceae |
| 371 | Bacteria; Firmicutes; Clostridia; Clostridiales; Ruminococcaceae; *Oscillospira* |
| 372 | Bacteria; Firmicutes; Clostridia; Clostridiales; |
| 373 | Bacteria; Firmicutes; Clostridia; Clostridiales; Lachnospiraceae |
| 374 | Bacteria; Firmicutes; Clostridia; Clostridiales; Ruminococcaceae |
| 375 | Bacteria; Firmicutes; Clostridia; Clostridiales; Lachnospiraceae |
| 376 | Bacteria; Firmicutes; Clostridia; Clostridiales; Ruminococcaceae |
| 377 | Bacteria; Firmicutes; Bacilli; Lactobacillales; Lactobacillaceae |
| 378 | Bacteria; Bacteroidetes; Bacteroidia; Bacteroidales |
| 379 | Bacteria; Firmicutes; Clostridia; Clostridiales; Lachnospiraceae |
| 380 | Bacteria; Firmicutes; Clostridia; Clostridiales; |
| 381 | Bacteria; Firmicutes; Clostridia; Clostridiales; Lachnospiraceae |
| 382 | Bacteria; Proteobacteria |
| 383 | Bacteria; Firmicutes; Clostridia |
| 384 | Bacteria; Proteobacteria; Deltaproteobacteria |
| 385 | Bacteria; Firmicutes; Clostridia |
| 386 | Bacteria; Firmicutes; Clostridia; Clostridiales; Lachnospiraceae |
| 387 | Bacteria; Firmicutes; Clostridia; Clostridiales; Lachnospiraceae |
| 388 | Bacteria; Firmicutes; Clostridia; Clostridiales; Lachnospiraceae |
| 389 | Bacteria; Firmicutes; Clostridia; Clostridiales |
| 390 | Bacteria; Proteobacteria; Epsilonproteobacteria; Campylobacterales; Campylobacteraceae; *Campylobacter* |
| 391 | Bacteria; Firmicutes; Clostridia |
| 392 | Bacteria; Firmicutes; Clostridia; Clostridiales; Ruminococcaceae |
| 393 | Bacteria; Firmicutes; Clostridia; Clostridiales; Lachnospiraceae |
| 394 | Bacteria; Firmicutes; Clostridia; Clostridiales; Lachnospiraceae |
| 395 | Bacteria; Firmicutes; Bacilli; Bacillales; Paenibacillaceae; *Brevibacillus* |
| 396 | Bacteria; Firmicutes; Clostridia; Clostridiales; Lachnospiraceae |
| 397 | Bacteria; Firmicutes; Clostridia; Clostridiales; Lachnospiraceae |
| 398 | Bacteria; Firmicutes; Clostridia; Clostridiales; Lachnospiraceae |
| 399 | Bacteria; Bacteroidetes; Bacteroidia; Bacteroidales |
| 400 | Bacteria; Firmicutes; Clostridia; Clostridiales |
| 401 | Bacteria; Firmicutes; Clostridia |
| 402 | Bacteria; Bacteroidetes; Sphingobacteriia; Sphingobacteriales; Ekhidnaceae |
| 403 | Bacteria; Firmicutes; Clostridia; Clostridiales; Ruminococcaceae; *Oscillospira* |
| 404 | Bacteria; Firmicutes; Clostridia; Clostridiales |
| 405 | Bacteria; Firmicutes; Clostridia; Clostridiales; Lachnospiraceae |
| 406 | Bacteria; Bacteroidetes; Bacteroidia; Bacteroidales |
| 407 | Bacteria |
| 408 | Bacteria; Firmicutes; Clostridia; Clostridiales |
| 409 | Bacteria; Firmicutes; Clostridia; Clostridiales; Ruminococcaceae |
| 410 | Bacteria; Firmicutes; Clostridia; Clostridiales; Lachnospiraceae |
| 411 | Bacteria; Tenericutes; Mollicutes |
| 412 | Bacteria; Firmicutes; Clostridia; Clostridiales; Lachnospiraceae |
| 413 | Bacteria; Firmicutes; Clostridia; Clostridiales; Lachnospiraceae |
| 414 | Bacteria; Firmicutes; Clostridia; Clostridiales; Lachnospiraceae |
| 415 | Bacteria; Firmicutes; Clostridia; Clostridiales; Ruminococcaceae |
| 416 | Bacteria; Firmicutes; Clostridia; Clostridiales; Lachnospiraceae |
| 417 | Bacteria; Firmicutes; Clostridia; Clostridiales; Lachnospiraceae |
| 418 | Bacteria; Firmicutes; Clostridia |
| 419 | Bacteria; Firmicutes; Clostridia; Clostridiales; Lachnospiraceae |
| 420 | Bacteria; Firmicutes; Clostridia; Clostridiales; Lachnospiraceae |
| 421 | Bacteria; Firmicutes; Clostridia; Clostridiales; Lachnospiraceae |
| 422 | Bacteria; Firmicutes; Clostridia; Clostridiales; Lachnospiraceae |
| 423 | Bacteria; Firmicutes; Clostridia; Clostridiales; Ruminococcaceae; *Ruminococcus* |
| 424 | Bacteria; Bacteroidetes; Flavobacteriia; Flavobacteriales; Flavobacteriaceae; *Flavobacterium* |
| 425 | Bacteria; Firmicutes; Clostridia; Clostridiales; Ruminococcaceae |
| 426 | Bacteria; Firmicutes; Clostridia; Clostridiales; Ruminococcaceae |
| 427 | Bacteria |
| 428 | Bacteria; Firmicutes; Clostridia; Clostridiales; Lachnospiraceae |
| 429 | Bacteria; Firmicutes; Clostridia; Clostridiales; Lachnospiraceae |
| 430 | Bacteria; Proteobacteria; Alphaproteobacteria; Rickettsiales; Pelagibacteraceae |
| 431 | Bacteria; Firmicutes; Clostridia; Clostridiales; Ruminococcaceae |
| 432 | Bacteria; Firmicutes; Clostridia; Clostridiales; Ruminococcaceae; *Oscillospira* |
| 433 | Bacteria; Firmicutes; Clostridia; Clostridiales; Lachnospiraceae |
| 434 | Bacteria; Firmicutes; Clostridia |
| 435 | Bacteria; Firmicutes; Clostridia; Clostridiales; Lachnospiraceae |
| 436 | Bacteria; Firmicutes; Bacilli; Bacillales; Staphylococcaceae; *Staphylococcus* |
| 437 | Bacteria; Firmicutes; Clostridia; Clostridiales; Lachnospiraceae |
| 438 | Bacteria; Firmicutes; Clostridia; Clostridiales; Lachnospiraceae |
| 439 | Bacteria; Firmicutes; Clostridia; Clostridiales; Lachnospiraceae |
| 440 | Bacteria; Firmicutes; Clostridia; Clostridiales; Ruminococcaceae; *Ruminococcus* |
| 441 | Bacteria; Firmicutes; Clostridia; Clostridiales; Lachnospiraceae |
| 442 | Bacteria; Firmicutes; Clostridia; Clostridiales; Ruminococcaceae |
| 443 | Bacteria; Firmicutes; Clostridia; Clostridiales; Ruminococcaceae; *Oscillospira* |
| 444 | Bacteria; Firmicutes; Clostridia; Clostridiales; Lachnospiraceae |
| 445 | Bacteria; Firmicutes; Clostridia; Clostridiales; Lachnospiraceae |
| 446 | Bacteria; Firmicutes; Clostridia; Clostridiales; Lachnospiraceae |
| 447 | Bacteria; Firmicutes; Clostridia; Clostridiales; Ruminococcaceae; *Oscillospira* |
| 448 | Bacteria; Firmicutes; Clostridia; Clostridiales; Lachnospiraceae |
| 449 | Bacteria; Firmicutes; Clostridia; Clostridiales; Lachnospiraceae |
| 450 | Bacteria; Firmicutes; Clostridia; Clostridiales; Lachnospiraceae; [*Ruminococcus*] *gnavus* |
| 451 | Bacteria; Proteobacteria; Gammaproteobacteria; Oceanospirillales; Halomonadaceae; *Candidatus Portiera* |
| 452 | Bacteria; Firmicutes; Clostridia; Clostridiales; Lachnospiraceae |
| 453 | Bacteria; Firmicutes; Clostridia |
| 454 | Bacteria; Bacteroidetes; Bacteroidia; Bacteroidales; Prevotellaceae; *Prevotella* |
| 455 | Bacteria; Firmicutes; Clostridia; Clostridiales; Ruminococcaceae |
| 456 | Bacteria; Firmicutes; Clostridia; Clostridiales; Ruminococcaceae; *Oscillospira* |
| 457 | Bacteria |
| 458 | Bacteria; Firmicutes; Clostridia; Clostridiales; Lachnospiraceae |
| 459 | Bacteria; Firmicutes; Clostridia; Clostridiales; Ruminococcaceae |
| 460 | Bacteria; Firmicutes; Clostridia; Clostridiales; Ruminococcaceae; *Oscillospira* |
| 461 | Bacteria; Firmicutes; Clostridia; Clostridiales; Lachnospiraceae |
| 462 | Bacteria; Firmicutes; Clostridia; Clostridiales; Lachnospiraceae |
| 463 | Bacteria; Firmicutes; Clostridia; Clostridiales; Lachnospiraceae |
| 464 | Bacteria; Firmicutes; Clostridia; Clostridiales; Ruminococcaceae |
| 465 | Bacteria; Firmicutes; Clostridia; Clostridiales; Lachnospiraceae |
| 466 | Bacteria; Firmicutes; Clostridia; Clostridiales; Clostridiaceae; *Clostridium* |
| 467 | Bacteria; Firmicutes; Bacilli; Lactobacillales; Lactobacillaceae |
| 468 | Bacteria; Firmicutes; Clostridia; Clostridiales; Lachnospiraceae |
| 469 | Bacteria; Firmicutes; Clostridia; Clostridiales; Lachnospiraceae |
| 470 | Bacteria; Firmicutes; Clostridia; Clostridiales; Ruminococcaceae; *Oscillospira* |
| 471 | Bacteria; Firmicutes; Clostridia; Clostridiales; Lachnospiraceae |
| 472 | Bacteria; Firmicutes; Clostridia; Clostridiales; Lachnospiraceae |
| 473 | Bacteria |
| 474 | Bacteria; Firmicutes; Clostridia; Clostridiales; Lachnospiraceae |
| 475 | Bacteria; Firmicutes |
| 476 | Bacteria; Firmicutes; Clostridia; Clostridiales; Ruminococcaceae |
| 477 | Bacteria; Firmicutes; Clostridia; Clostridiales; Peptostreptococcaceae |
| 478 | Bacteria; Firmicutes; Clostridia; Clostridiales; Lachnospiraceae |
| 479 | Bacteria; Firmicutes; Clostridia; Clostridiales; Lachnospiraceae |
| 480 | Bacteria; Firmicutes; Clostridia; Clostridiales; Lachnospiraceae |
| 481 | Bacteria; Firmicutes; Clostridia; Clostridiales; Lachnospiraceae |
| 482 | Bacteria; Firmicutes; Clostridia; Clostridiales; |
| 483 | Bacteria; Firmicutes; Clostridia; Clostridiales; Lachnospiraceae |
| 484 | Bacteria; Firmicutes; Clostridia; Clostridiales; Lachnospiraceae |
| 485 | Bacteria |
| 486 | Bacteria; Firmicutes; Clostridia |
| 487 | Bacteria; Proteobacteria; Gammaproteobacteria; Xanthomonadales; Xanthomonadaceae; *Stenotrophomonas* |
| 488 | Bacteria; Firmicutes; Clostridia; Clostridiales; Lachnospiraceae |
| 489 | Bacteria; Firmicutes; Clostridia; Clostridiales; Lachnospiraceae; [*Ruminococcus*] *gnavus* |
| 490 | Bacteria; Firmicutes; Clostridia; Clostridiales; Lachnospiraceae; [*Ruminococcus*] *gnavus* |
| 491 | Bacteria; Firmicutes; Clostridia; Clostridiales; Lachnospiraceae |
| 492 | Bacteria; Tenericutes; Mollicutes |
| 493 | Bacteria; Proteobacteria; Betaproteobacteria; Burkholderiales; Comamonadaceae; *Delftia* |
| 494 | Bacteria; Firmicutes; Clostridia; Clostridiales; Lachnospiraceae |
| 495 | Bacteria; Firmicutes; Clostridia; Clostridiales; Lachnospiraceae |
| 496 | Bacteria; Firmicutes; Clostridia; Clostridiales; Lachnospiraceae |
| 497 | Bacteria; Firmicutes; Clostridia; Clostridiales; Ruminococcaceae; *Oscillospira* |
| 498 | Bacteria; Firmicutes; Clostridia; Clostridiales; Lachnospiraceae |
| 499 | Bacteria; Firmicutes; Clostridia |
| 500 | Bacteria; Firmicutes; Clostridia; Clostridiales; Ruminococcaceae |
| 501 | Bacteria; Firmicutes; Clostridia; Clostridiales; Ruminococcaceae; *Oscillospira* |
| 502 | Bacteria; Proteobacteria; Alphaproteobacteria; Rickettsiales |
| 503 | Bacteria; Bacteroidetes; Bacteroidia; Bacteroidales |
| 504 | Bacteria; Actinobacteria; Actinobacteria; Actinomycetales; Nocardiopsaceae |
| 505 | Bacteria; Firmicutes; Clostridia; Clostridiales; Lachnospiraceae |
| 506 | Bacteria; Firmicutes; Clostridia |
| 507 | Bacteria; Firmicutes; Clostridia; Clostridiales |
| 508 | Bacteria; Firmicutes; Clostridia; Clostridiales; Lachnospiraceae |
| 509 | Bacteria; Firmicutes; Clostridia; Clostridiales; Ruminococcaceae; *Anaerotruncus* |
| 510 | Bacteria |
| 511 | Bacteria; Firmicutes; Clostridia; Clostridiales; Lachnospiraceae |
| 512 | Bacteria; Firmicutes; Clostridia; Clostridiales; |
| 513 | Bacteria; Firmicutes; Clostridia; Clostridiales; Ruminococcaceae; *Oscillospira* |
| 514 | Bacteria; Firmicutes; Clostridia; Clostridiales; Lachnospiraceae |
| 515 | Bacteria; Firmicutes; Erysipelotrichi; Erysipelotrichales; Erysipelotrichaceae |
| 516 | Bacteria; Firmicutes; Clostridia; Clostridiales; Lachnospiraceae |
| 517 | Bacteria; Firmicutes; Clostridia; Clostridiales; Lachnospiraceae |
| 518 | Bacteria; Firmicutes; Clostridia; Clostridiales; Ruminococcaceae; *Oscillospira* |
| 519 | Bacteria; Proteobacteria; Gammaproteobacteria; Pseudomonadales; Pseudomonadaceae |
| 520 | Bacteria |
| 521 | Bacteria; Firmicutes; Clostridia |
| 522 | Bacteria; Proteobacteria; Gammaproteobacteria; Pseudomonadales; Pseudomonadaceae; *Pseudomonas viridiflava* |
| 523 | Bacteria; Firmicutes; Clostridia; Clostridiales; Ruminococcaceae; *Ruminococcus* |
| 524 | Bacteria; Firmicutes; Clostridia; Coriobacteriales; Coriobacteriaceae; *Adlercreutzia* |
| 525 | Bacteria; Firmicutes; Clostridia; Clostridiales |
| 526 | Bacteria |
| 527 | Bacteria; Firmicutes; Clostridia; Clostridiales; Lachnospiraceae |
| 528 | Bacteria; Firmicutes; Clostridia; Clostridiales; Lachnospiraceae |
| 529 | Bacteria; Firmicutes; Clostridia; Clostridiales; Lachnospiraceae |
| 530 | Bacteria; Firmicutes; Clostridia |
| 531 | Bacteria; Firmicutes; Clostridia; Clostridiales; Ruminococcaceae |
| 532 | Bacteria; Firmicutes; Clostridia; Clostridiales; Lachnospiraceae |
| 533 | Bacteria; Firmicutes; Clostridia; Clostridiales; Ruminococcaceae |
| 534 | Bacteria; Firmicutes; Clostridia; Clostridiales; Ruminococcaceae |
| 535 | Bacteria; Firmicutes; Clostridia; Clostridiales; Lachnospiraceae |
| 536 | Bacteria; Firmicutes; Clostridia |
| 537 | Bacteria; Firmicutes; Clostridia; Clostridiales; Ruminococcaceae |
| 538 | Bacteria; Firmicutes; Clostridia; Clostridiales; Ruminococcaceae; *Oscillospira* |
| 539 | Bacteria; Firmicutes; Clostridia; Clostridiales; Lachnospiraceae |
| 540 | Bacteria; Firmicutes; Clostridia |
| 541 | Bacteria; Firmicutes; Clostridia; Clostridiales; Lachnospiraceae |
| 542 | Bacteria; Firmicutes; Clostridia; Clostridiales; Lachnospiraceae |
| 543 | Bacteria; Firmicutes; Clostridia; Clostridiales; Ruminococcaceae |
| 544 | Bacteria; Firmicutes; Clostridia; Clostridiales; Lachnospiraceae |
| 545 | Bacteria; Firmicutes; Clostridia; Clostridiales |
| 546 | Bacteria; Firmicutes; Clostridia; Clostridiales; Lachnospiraceae |
| 547 | Bacteria; Firmicutes; Clostridia |
| 548 | Bacteria; Firmicutes; Clostridia; Clostridiales; Lachnospiraceae |
| 549 | Bacteria; Firmicutes; Clostridia; Clostridiales; Clostridiaceae |
| 550 | Bacteria; Firmicutes; Clostridia; Clostridiales; Lachnospiraceae |
| 551 | Bacteria; Firmicutes; Clostridia; Clostridiales; Lachnospiraceae |
| 552 | Bacteria; Firmicutes; Clostridia; Clostridiales |
| 553 | Bacteria; Bacteroidetes; Bacteroidia; Bacteroidales |
| 554 | Bacteria; Bacteroidetes; Bacteroidia; Bacteroidales |
| 555 | Bacteria; Firmicutes |
| 556 | Bacteria; Firmicutes; Clostridia |
| 557 | Bacteria; Firmicutes; Erysipelotrichi; Erysipelotrichales; [Coprobacillaceae]; *Coprobacillus* |
| 558 | Bacteria; Firmicutes; Clostridia; Clostridiales; Lachnospiraceae; [*Ruminococcus*] *gnavus* |
| 559 | Bacteria; Firmicutes; Clostridia |
| 560 | Bacteria; Firmicutes; Clostridia; Clostridiales; Ruminococcaceae |
| 561 | Bacteria |
| 562 | Bacteria; Firmicutes; Clostridia; Clostridiales; Lachnospiraceae |
| 563 | Bacteria; Firmicutes; Clostridia; Clostridiales; Lachnospiraceae |
| 564 | Bacteria; Firmicutes; Clostridia; Clostridiales; Ruminococcaceae; *Oscillospira* |
| 565 | Bacteria; Firmicutes; Clostridia; Clostridiales; Lachnospiraceae |
| 566 | Bacteria; Firmicutes; Clostridia; Clostridiales; Lachnospiraceae |
| 567 | Bacteria; Firmicutes; Clostridia; Clostridiales; Ruminococcaceae; *Oscillospira* |
| 568 | Bacteria; Firmicutes; Clostridia; Clostridiales; Ruminococcaceae; *Anaerotruncus* |
| 569 | Bacteria; Firmicutes; Clostridia |
| 570 | Bacteria; Firmicutes; Clostridia; Clostridiales; Lachnospiraceae |
